# Death agonist antibody against TRAILR2/DR5/TNFRSF10B enhances birinapant anti-tumor activity in HPV-positive head and neck squamous cell carcinomas

**DOI:** 10.1038/s41598-021-85589-5

**Published:** 2021-03-18

**Authors:** Yi An, Jun Jeon, Lillian Sun, Adeeb Derakhshan, Jianhong Chen, Sophie Carlson, Hui Cheng, Christopher Silvin, Xinping Yang, Carter Van Waes, Zhong Chen

**Affiliations:** 1 Tumor Biology Section, Head and Neck Surgery Branch, National Institute on Deafness and Other Communication Disorders, National Institutes of Health, Building 10, 7N240, Bethesda, MD 201892 USA; 2 NIH Medical Research Scholars Program, Bethesda, MD USA

**Keywords:** Cancer, Drug discovery

## Abstract

Head and neck squamous cell carcinomas (HNSCC) induced by human papillomavirus (HPV) have increased recently in the US. However, the distinct alterations of molecules involved in the death pathways and drug effects targeting inhibitor of apoptosis proteins (IAPs) have not been extensively characterized in HPV(+) HNSCC cells. In this study, we observed the distinct genomic and expression alterations of nine genes involved in cell death in 55% HNSCC tissues, which were associated with HPV status, tumor staging, and anatomic locations. Expression of four genes was statistically correlated with copy number variation. A panel of HPV(+) HNSCC lines showed abundant TRAILR2 and IAP1 protein expression, but were not sensitive to IAP inhibitor birinapant alone, while combinatory treatment with TNFα or especially TRAIL enhanced this drug sensitivity. The death agonistic TRAILR2 antibody alone showed no cell inhibitory effects, whereas its combination with birinapant and/or TRAIL protein demonstrated additive or synergistic effects. We observed predominantly late apoptosis mode of cell death after combinatorial treatments, and pan-caspase (ZVAD) and caspase-8 (ZIETD) inhibitors attenuated treatment-induced cell death. Our genomic and expression data-driven study provides a framework for identifying relevant combinatorial therapies targeting death pathways in HPV(+) HNSCC and other squamous cancer types.

## Introduction

A critical step in cancer development is to escape the self-regulated program of apoptotic cell death and differentiation, despite retained expression of molecules involved in the death signaling pathways^1–5^. In normal human cells, there are two primary pathways that trigger apoptosis: the extrinsic and intrinsic pathways. The former begins with interaction between the death ligands, such as tumor necrosis factor (TNFα) and TNF-related apoptosis inducing ligand (TRAIL, also called TNFSF10, Tumor Necrosis Factor Ligand Superfamily 10), and their corresponding receptors^5–7^. The intrinsic pathway begins with stress-induced mitochondrial permeabilization leading to the release of cytochrome complex (cytochrome *c*) and second mitochondria-derived activator of caspases (SMAC)^3,8,9^. Recently, The Cancer Genome Atlas (TCGA) project of head and neck squamous cell carcinomas (HNSCC) revealed that 44% human papilloma virus negative (HPV−) and 31% HPV(+) patients exhibited genetic or expression alterations of molecules involved in the death signaling pathways^10^. Furthermore, through the PanCancer Atlas study of five squamous cell carcinomas (SCCs), including head and neck, lung, esophagus, cervix, and bladder, we have identified deregulation of various components of the extrinsic and intrinsic apoptotic pathways, such as alterations in gene expression and genomic amplification of the Fas-associated death domain (FADD) and inhibitor of apoptosis proteins (IAPs)^11^. HPV(−) HNSCC and other SCC tumors commonly contain co-amplification of chromosome 11q13, harboring the FADD gene, and 11q22, where BIRC2 and BIRC3 genes encode cIAP1 and cIAP2 proteins, respectively. FADD and IAPs are important downstream signaling molecules involved in the extrinsic death pathways, while XIAP is also involved in the intrinsic pathway^5,12–14^. However, it is still not well understood how alterations in the expression of FADD and IAPs affect death pathway signaling and contribute to resistance to therapies targeting the death pathways in cancers, especially in HNSCC differing in HPV status.

Recent drug development efforts have been aimed at targeting the extrinsic death pathways, especially through the TRAIL receptors. Humanized monoclonal antibody agonists to TRAILR1 (DR4) and TRAILR2 (DR5) offer longer in vivo half-life and higher specificity against the death receptors compared to TRAIL^6,15^. In addition, the antibodies could trigger antibody-dependent cell-mediated cytotoxicity (ADCC) and complement-dependent cellular cytotoxicity (CDC), through interactions of the antibody Fc domains with Fc receptors on the cell surface of immune cells, which provide additional death signaling and immune recruitment to eradicate cancer cells^16,17^. Despite the promising biological mechanism and relevant preclinical testing, clinical trials with these antibodies have shown lack of clinical efficacy, whether used alone or in combination with chemotherapy^6,15,18^. It has been hypothesized that the failed clinical trials using the antibodies targeting TRAILRs could be due to the resistance of primary tumors to apoptosis, the mechanism for which remains unclear.

In parallel to the development of antibodies targeting the TRAILRs, small molecule inhibitors have been identified to target the molecules involved in the downstream components of the death pathways. Birinapant is a novel bivalent peptidomimetic of the second mitochondria-derived activator of caspases (SMAC), which is an endogenous protein released by mitochondria involved in the intrinsic death pathway^19,20^. Birinapant is capable of inducing cancer cell death through SMAC mimetic by degrading IAPs through binding, preferentially targeting cIAP1 compared to cIAP2 and XIAP^21,22^. Recently, we have investigated the anti-tumor effects of birinapant in HPV(−) HNSCC cells, and observed that cells retaining FADD/BIRC2 amplification and overexpression were sensitive to birinapant, and the effects were enhanced by death agonists TNFα or TRAIL^23^. Intriguingly, the single HPV(+) HNSCC line included in the panel was sensitized by birinapant to TRAIL to a greater extent than TNFα. Together with TCGA data showing that FADD amplification and overexpression implicated in sensitivity to the combination is not commonly observed in HPV(+) HNSCC, these observations raised the question whether the SMAC mimetic birinapant could be used in combination with TRAIL or TRAIL agonists for treating this type of cancer.

To address these questions, we characterized the genomic and expression profiles of nine molecules involved in the extrinsic and intrinsic death pathways, including TRAIL (TNFSF10) and its receptors, FADD, BIRC2/3, and XIAP in HNSCC tissues differing in HPV status from TCGA datasets. We observed a distinct pattern of genomic and expression alterations of those molecules with respect to the HPV status. We found that HPV(+) HNSCC cells which harbor less frequent FADD amplification and overexpression, and with more frequent shallow deletion of BIRC2/3 loci, are less sensitive to the SMAC mimetic birinapant. In addition, we observed that HPV(+) HNSCC cells exhibited genomic gain and/or overexpression of TRAIL and TRAILRs, and the stimulation of the death pathway through anti-TRAILR2/DR5 antibody agonist was very effective in inducing cell death in combination with birinapant and/or TRAIL. This study utilized the available genomic and genetic expression profile of HNSCC to develop a mechanism and data-driven hypothesis. This led to experimental support for the incorporation of birinapant, anti-TRAILR antibody, and TRAIL for the treatment of HPV(+) HNSCC cells that were otherwise insensitive to IAP inhibitor birinapant alone.

## Results

### Altered genomic and gene expression profile in cell death pathways of HNSCC differing in HPV status from TCGA

**Figure 1:**
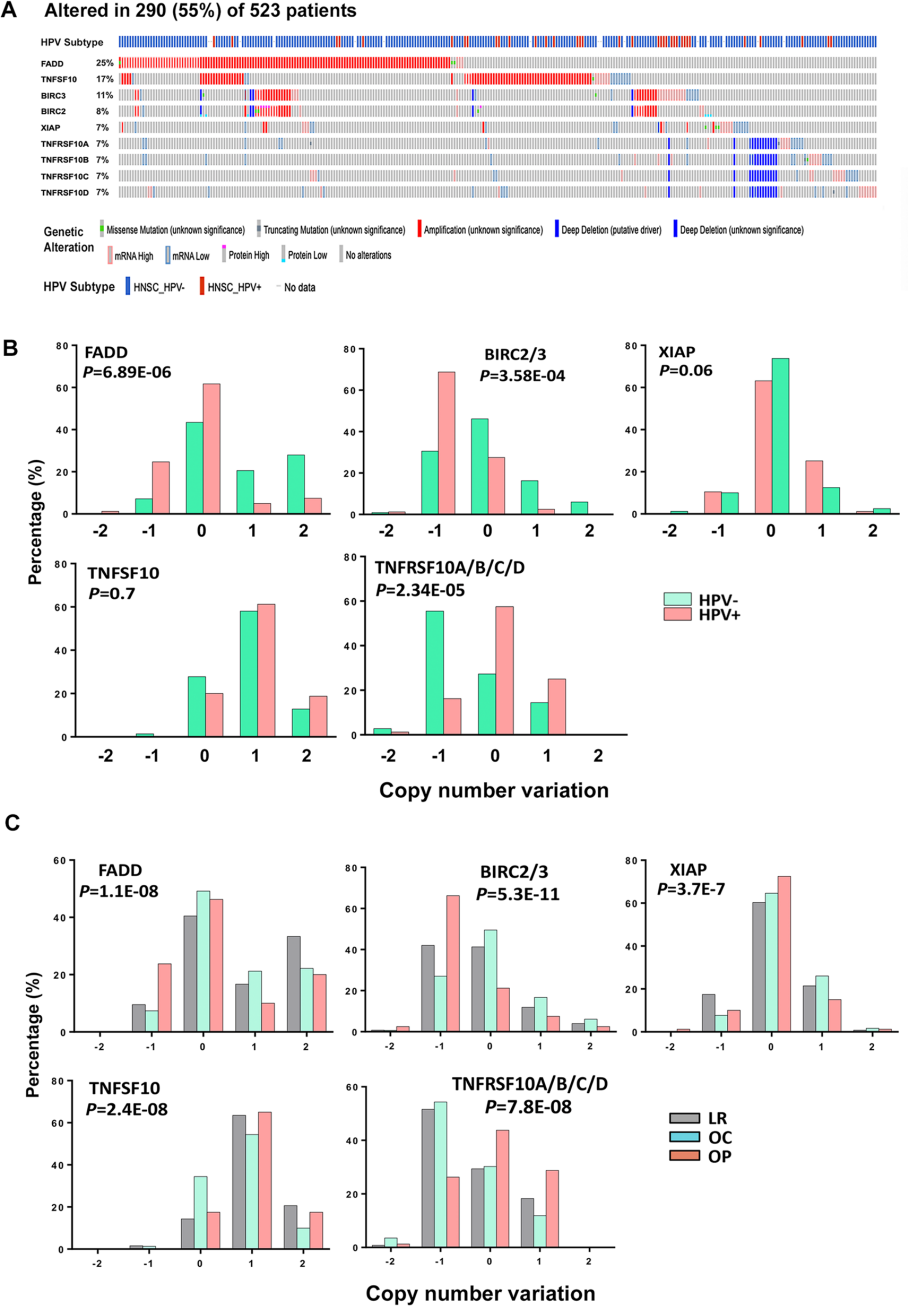
Genetic and expression alteration of genes involved in cell death pathways from HNSCC TCGA dataset. (**A**) 523 HNSCC cases were analyzed using TCGA PanCancer Atlas dataset and presented in Oncoprint format using cBioPortal website. 290 (55%) samples exhibited genetic and expression alterations of the nine genes involved in the death pathway. The genetic alterations include equal or greater than two copy gain (amplification), two copy loss (deep deletion), and truncating and missense mutations. Percentage of each gene’s alteration in total patient samples was represented on the left, and each bar represents an individual patient sample. The blue bar at the top: HPV(−) samples, and the red bar: HPV(+) samples. The primary tumor sites: larynx: blue; oral cavity: red; oropharynx: orange; hypopharynx: green. (**B**) The genes with statistical significance in distribution of various CNV between HPV(−) samples (green bar) and HPV(+) samples (red bar). CNV were analyzed by GISTC and presented in x axis, as two copy DNA loss [homozygous deletion, − 2], single copy loss [heterozygous deletion, − 1], diploid (0), one copy gain (1), and amplification (two copy gain or more, 2). The percentage of each CNV types in their respective HPV status groups were calculated based on the HNSCC sample counts. (**C**) CNV among different primary tissue sites were examined and analyzed as in (**B**). The primary tumor site, larynx (LR): gray; oral cavity (OC): blue; oropharynx (OP): red. Statistical analysis was conducted by Fisher exact test.

FADD, IAPs, and TRAIL family components involved in the cell death pathways frequently exhibit genomic and gene expression alterations^7,11^. However, the differences in the genomic and expression alterations between HPV(+) and (−) tumor types have not been fully elucidated in the latest HNSCC TCGA dataset [523 total cases with 80 HPV(+), 434 HPV(−) cases, and 9 of unknown status]^11^. Prominent genetic changes, such as mutations, gene amplifications, and deep deletions (two-copy loss), as well as altered gene expressions are presented in Oncoprint (Fig. 1A). Among the nine genes analyzed, FADD was the most altered gene with a high percentage of amplifications and overexpression in 25% of total samples (Fig. 1A). The gene for TNFSF10 (TRAIL), a ligand for TRAIL receptor family members, was amplified and overexpressed in 17% patient samples. *BIRC2/3* and *XIAP* also showed gene amplification, and the deletion of TNFRSF10A/B/C/D (TRAIL receptors) were clustered together due to their genomic co-localization at chromosome 8p21.3 (Fig. 1A).

Next, we stratified the DNA copy number variations (CNV) for the death molecules and compared their distributions between HPV(+) and HPV(−) tumors (Fig. 1B). Both *FADD* and *BIRC2/3* show significant differences in CNV between HPV(+) and HPV(−) tumors. HPV(−) tumors exhibited higher percentages of overall *FADD* amplifications, whereas HPV(+) tumors showed a higher percentage of *BIRC2/3* single copy loss. The CNV components for XIAP and TNFSF10 exhibited less significant difference or similar distributions between tumors with different HPV status. The TRAIL receptor family members (TNFRSF10A/B/C/D) exhibited significant difference in CNV components between tumors with different HPV status, that HPV(−) tumors had the higher percentage of one-copy loss, and HPV(+) tumors more often displayed neutral or one copy gain (Fig. 1B). The chromosome view of CNV were compared for FADD, BIRC2/3, XIAP, TNFRSF10A/B/C/D genes in 80 HPV(+) HNSCC tissues from TCGA dataset and 11 HPV(+) HNSCC cell lines sequenced by our group in Supplemental Figure 1A–D.

Furthermore, we investigated CNV changes in distinct primary tumor sites of HNSCC, such as larynx (LR), oral cavity (OC), and oropharynx (OP). The genetic alterations of all the genes differed significantly among the primary tumor sites (Fig. 1C). Tumors from LR and OC are characterized by higher percentages of *FADD* one-copy gain compared to that of OP, and the amplification of *FADD* with two-copy gain is higher in LR only. OP tumors, enriched for HPV(+) HNSCC, showed the highest percentage of one-copy loss of *FADD* and *BIRC2/3*, similar to the distribution of CNV in HPV(+) tumors. One-copy loss of XIAP was also relatively higher for both HPV(+) and LR group compared to other groups. Various degrees of gene amplifications were observed in *TNFSF10 *(*TRAIL*) for the majority of tumors from all sites, and tumors from OP and LR exhibited relatively higher percentages of two-copy gene amplification. In contrast, more tumors from LR and OC were characterized by one-copy deletions of TNFRSF10A/B/C/D (TRAILRs), while HPV(+) tumors from OP exhibited higher frequency of one-copy gain. The high percentage loss of *FADD* and *BIRC2/3* and gain in *TRAIL* and receptors in HPV(+) OP tumors support our hypothesis that these subsets of tumors could differ in sensitivity to birinapant and agents targeting TRAILRs.

We next examined the genetic alterations of *TP53* and *CASP8*, which are important molecules that modulate the cell apoptosis pathway^5,24,25^. The genetic alterations of *TP53* and *CASP8* from HNSCC TCGA datasets were displayed by Oncoprint, which showed 71% *TP53* and 13% *CASP8* mutation rates, mainly in HPV(−) HNSCC (Supplemental Figure 2A). Among this cohort containing 80 HPV(+) cases, there are only 7 cases with *TP53* mutation, and only one case with both *TP53* mutation and *CASP8* amplification. Interestingly, the genetic alterations of *TP53* and *CASP8* exhibited statistically significant mutual exclusivity (Supplemental Figure 2B). The data suggests that *TP53* and *CASP8* mutations are among the major anti-apoptosis mechanisms involved in HPV(−) HNSCC, whereas those involved in HPV(+) HNSCC are known to include viral inactivation of TP53.

### Genetic alterations of the death pathway in major cancer types from TCGA datasets

To explore the broader indications of these genetic alterations involved in the death pathway, we surveyed these gene status in 33 major cancer types with TCGA datasets in cBioPortal (https://www.cbioportal.org), and ranked the cancer types based on the frequency of CNV and mutations (Supplemental Figure 3A). Interestingly, lung squamous cell carcinoma (SCC) and esophageal cancer (most SCC) ranked as the top two cancer types over HNSCC, and the top six cancer types were all SCCs, except ovarian cancer (Supplemental Figure 3A). Most of the genetic alterations present are amplifications, consistent with those observed in HNSCC (Fig. 1). In lung SCC, amplification and overexpression of TNFSF10/TRAIL (46%) and FADD (18%) were the top genes observed, while deletion of TRAILRs were observed in 6–7% patient samples (Supplemental Figure 3B). Lung SCC patients with FADD amplification exhibited worse disease/progression-free survival, whereas patients with amplification or overexpression of TNFSF10/TRAIL had better survival (Supplemental Figure 3C,D). Together, these gene signatures support further investigation of the potential of targeted therapy of death pathways in SCCs.

### Association of mRNA expression with copy number alteration of the genes involved in the death pathway in HNSCC tissues and cell lines

**Figure 2:**
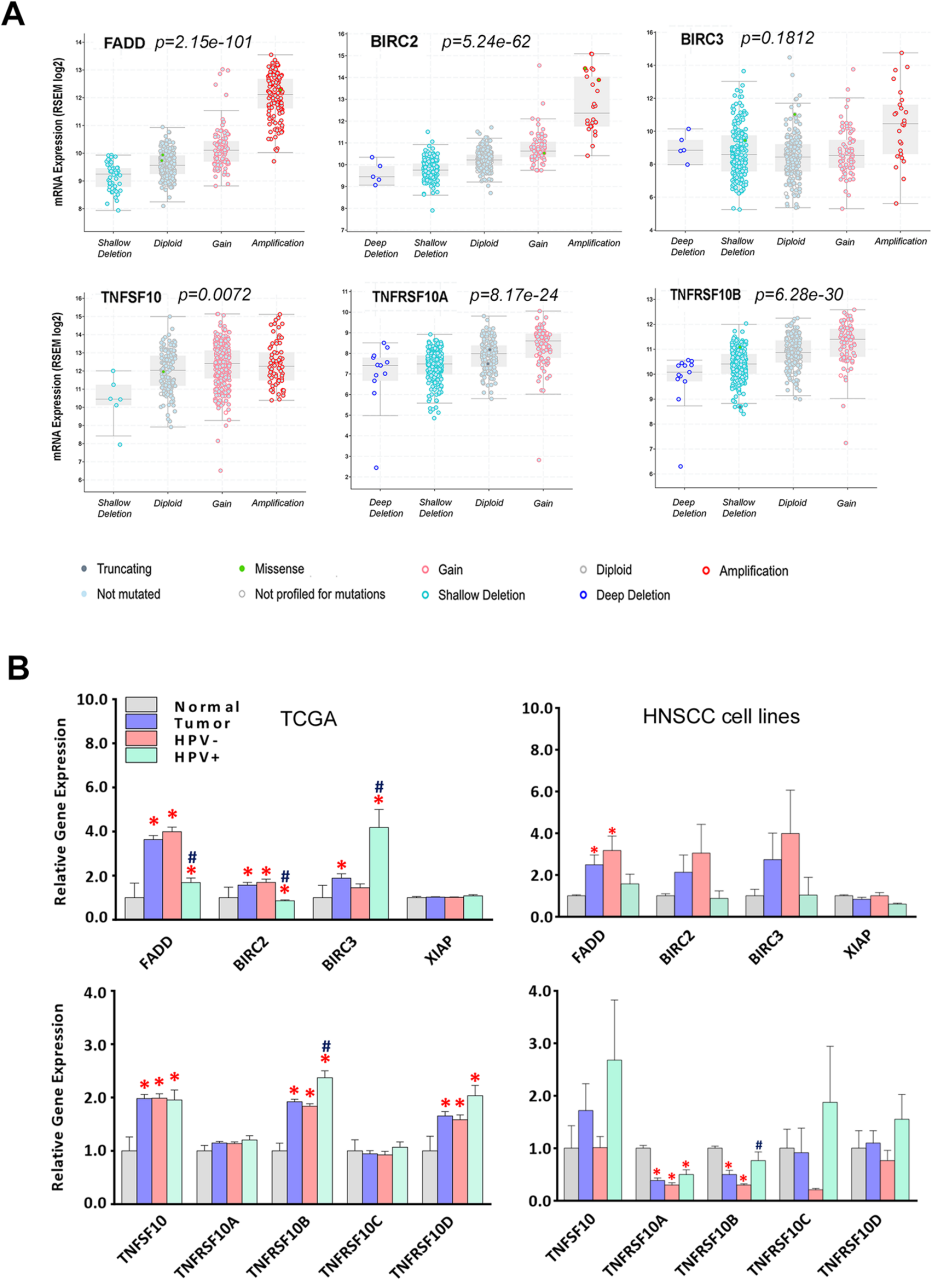
Association of copy number alterations with mRNA expression in HNSCC tumors and cell lines. (**A**) Expression and copy number status for six of nine genes are presented, including five with significant correlation between mRNA expression and DNA CN in 523 HNSCC tumor specimens from the TCGA data set. mRNA expression by RNAseq is presented in the y axis as the log2 scale. p-values present the significance of the Spearman correlation of mRNA expression as a function of CNV. (**B**) Expression of these genes in TCGA dataset (left) and HNSCC cell lines sequenced in our laboratory (right) are presented in a bar graph format, and the samples labeled as normal, tumor [HPV(−) plus HPV(+)], HPV(−), or HPV(+). The altered genes are presented by the relative gene expression levels as mean ± standard errors (SE). Statistical difference was calculated by Student’s *t* test, p < 0.05. * indicates statistical significances when compared with normal mean, and # indicates significance when compared samples different in HPV status.

We further analyzed the genetic basis of gene expression levels of the molecules involved in the death pathway using mRNA sequencing data from HNSCC TCGA cohorts (Fig. 2A). Among the nine genes examined, mRNA expression of *FADD*, *BIRC2*, and *TNFRSF10A/B* were strongly correlated with CNV with high statistical significance. In addition, we made comparisons in gene expression between tumors and their adjacent normal tissues, as well as between tumors with differing HPV status (Fig. 2B, left panels). In parallel, we also compared these gene expression levels using our RNA sequencing data from 26 HNSCC cell lines^26^ (Fig. 2B, right panels). From TCGA datasets, *FADD*, *BIRC2/3*, *TNFSF10*, and *TNFRSF10B/C/D* were overexpressed in tumor cases compared to normal. Interestingly, consistent with their CNV patterns, *FADD* and *BIRC2* expression levels were higher in HPV(−) tumors, while *BIRC3*, *TNFSF10*, and *TNFRSF10B/C/D* expression levels were higher in HPV(+) tumors (Fig. 2B, left panels). In HNSCC cell lines, *FADD* expression was higher in HPV(−) tumor lines, and *TNFRSF10A/B/C* exhibited decreased expression in HNSCC compared to normal human oral keratinocyte lines (HOK) (Fig. 2B, right panels). The difference between tumor tissues and cell lines could be due to the enrichment in HNSCC lines of chromosomal copy alterations typical of in more aggressive HPV(−) and HPV (+) tumors, as well as lacking tumor stromal cells in the culture condition^26^.

### FADD/BIRCs protein expression and TRAILR2/DR5/TNFRSF10B cell surface levels in HPV(+) cell lines

**Figure 3:**
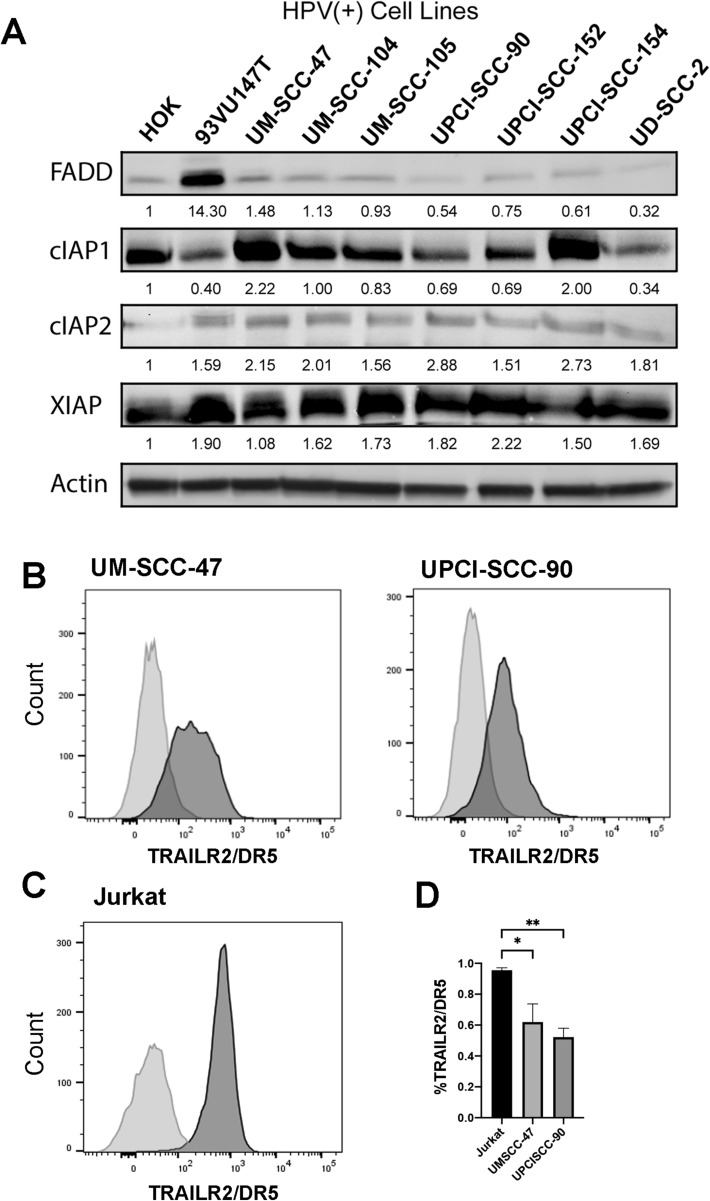
Protein expression of FADD, IAPs, and TRAILR2/DR5 in HPV(+) HNSCC cell lines. (**A**). Protein expression from whole cell lysates of HOK and a panel of HPV(+) HNSCC cell lines were examined by Western blot. Protein expression was quantified by comparison to HOK and actin as a loading control. (**B**) UM-SCC-47 and UPCI-SCC-90 cells were stained with fluorescent conjugated anti-TRAILR2/DR5 antibody and Zombie-violet for viability. The x-axis represents TRAILR2/DR5 fluorescent intensity, and the y-axis represents cell count. Percentages of TRAILR2/DR5 positivity among live cells for each cell line are represented and compared with the negative controls of unstained cells. (**C**) TRAILR2/DR5 staining of Jurkat cells was served as a positive control for data presented by (**B**), and the unstained cells served as the negative control. (**D**) There was no statistical difference in the percentages of TRAILR2/DR5 positive staining cells between the two HNSCC cell lines. The data are presented by six replicates from two independent experiments. * or ** indicates Student’s *t* test, p < 0.05 or 0.01.

Western blot analysis was used to determine baseline protein expression levels for FADD, cIAP1, cIAP2, and XIAP in eight different HPV(+) HNSCC cell lines compared to the HOK cell line (Fig. 3A, Supplemental Figure 4). FADD protein was overexpressed only in the 93VU147T cell line, which is consistent with our previous finding of a 11q13 amplification containing FADD gene in this line^26^. There was uniformly increased expression of cIAP1/2 and XIAP across all cell lines compared with the control HOK cells.

**Figure 4:**
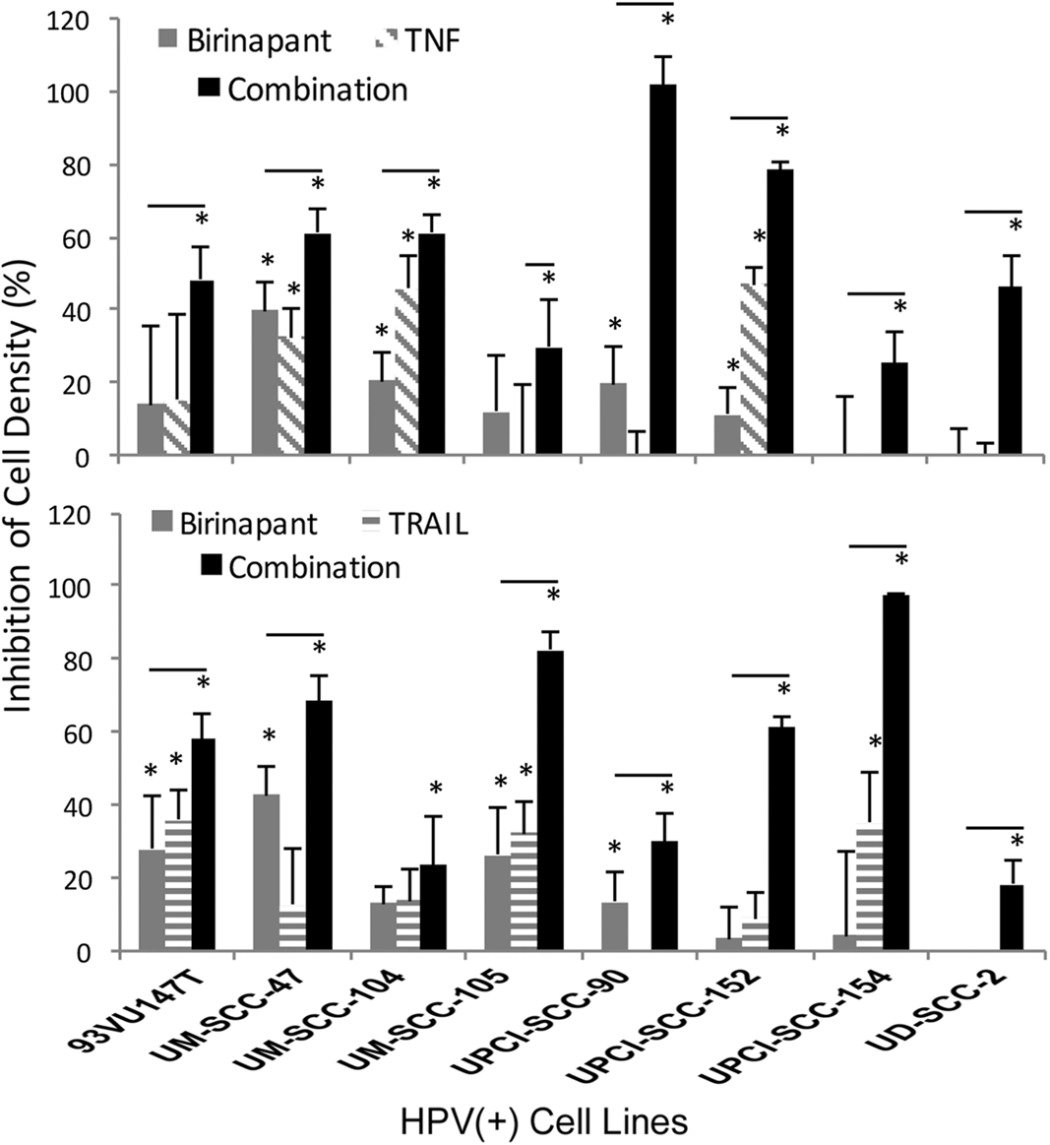
The anti-proliferative effects of birinapant, TNF, and TRAIL alone and in combination in HPV(+) cell lines. Effect of birinapant with and without death agonists TNFα or TRAIL were tested on 8 HPV(+) HNSCC cell lines. The top panel shows the percent inhibition assessed by XTT cell density assay on day 3 post treatment with 1 µM birinapant or 20 ng/mL TNFα alone or in combination. The bottom panel shows cell inhibition after treated with 1 µM birinapant or 50 ng/mL TRAIL alone or in combination. Independent experiments were performed to titrate the drug doses, the combination, and time points previously. Error bars, the standard deviation of 6 replicates are presented from a representative of independent experiments. Values were normalized to the untreated cells for the same experiment. Columns marked with * were statistically significantly different than the untreated control. Separately, a bar overlying the * indicates a statistically significant difference between combination treatment and the individual treatment(s) that the bar extends to. Student’s *t* test: *p < 0.05.

We examined two HPV(+) cell lines for their surface expression of TRAILR2/DR5/TNFRSF10B by immunofluorescent staining for flow cytometry analysis. From our panel of HPV(+) cell lines tested, UM-SCC-47 and UPCI-SCC-90 were chosen for further investigations based on their drug sensitivity (confirmed below in Fig. 4 and Supplemental Table 1). Both cell lines demonstrated TRAILR2/DR5 on cell surfaces with receptor staining compared to samples stained only with viability dye (Fig. 3B). The degree of TRAILR2/DR5 positivity among live cells was not different between UM-SCC-47 and UP-CISCC-90 cell lines, and both were lower in percentage when compared to the positive control of Jurkat cells (Student’s *t* test, p < 0.01) (Fig. 3C,D, Supplemental Figure 5).

### The anti-proliferative effects of birinapant, TNF, or TRAIL alone and in combination in HPV(+) cell lines

Next we examined the drug sensitivity of the HPV(+) cell lines. XTT cell density assay was conducted in cells treated with birinapant 1 μM alone or in combination with death agonists TNFα 20 ng/mL or TRAIL 50 ng/mL. Preliminary experiments were conducted to determine the IC_50_ values for birinapant alone and in conjunction with TNFα or TRAIL (Supplemental Table 1). All cell lines failed to reach IC_50_ when treated with birinapant up to 5 μM for 3 days, and only two reached the threshold by day 5. However, most of the lines achieved or exceeded IC_50_ by day 3 or 5 when treated with birinapant combined with either TNFα or TRAIL. The extent of inhibition of cellular density with birinapant alone and in combination with TNFα or TRAIL observed at 3 days (Fig. 4) and 5 days post-treatment (Supplemental Figure 6). As observed previously, UM-SCC-47 and some HPV(+) lines showed greater inhibition with birinapant in combination with TRAIL compared to TNFα, while others were relatively more sensitive to TNFα at the concentrations studied. Overall, a marked enhancement in inhibitory activity was noted with the combinations across nearly all cell lines at both time points.

### Anti-TRAILR2 (DR5) antibody enhanced the effects of birinapant alone and in combination with TNF-α or TRAIL in HPV(+) HNSCC cell lines

**Figure 5:**
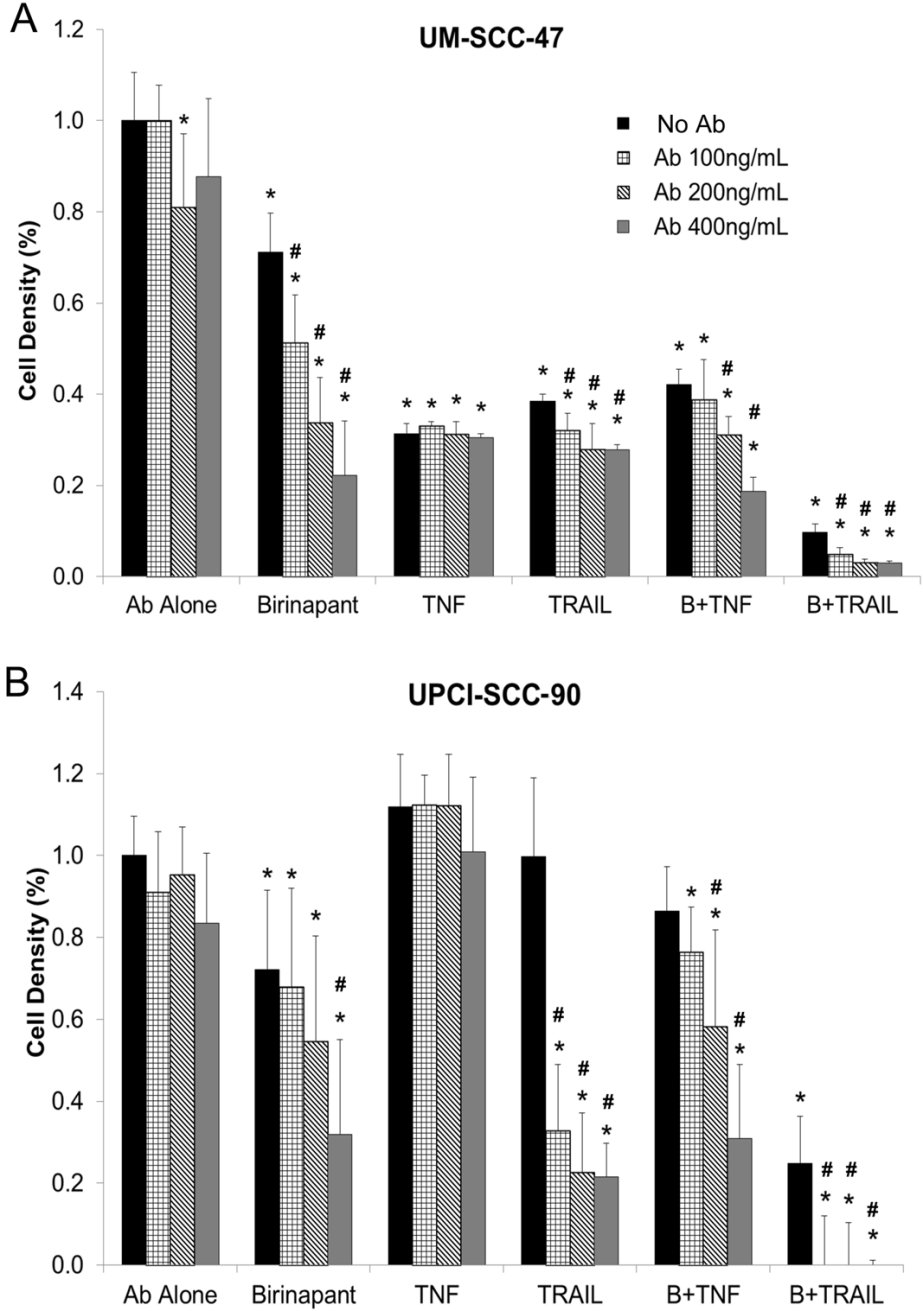
Antiproliferative effect of birinapant is enhanced by TRAIL, TRAILR2 antibody, and TNF-α in combination treatment. Inhibition of cell density was measured by XTT after administering birinapant, TRAIL, TNF-α, TRAILR2 antibody alone and in combination on HPV(+) cell lines UM-SCC-47 cells (**A**, day 3), and UPCI-SCC-90 (**B**, day 5). Independent experiments were performed to titrate the drug doses, the combination, and different time points previously. Error bars, the standard deviation of 6 replicates from a representative of independent experiments. * denotes a significant p-value when compared with no treatment control, the first column. # denotes a significant p-value when compared with no antibody control of each treatment group. p < 0.05 by Student’s *t* test.

Based on our observation that TNFRSF10A/B/C/D (TRAILRs) were rarely deleted and their expression levels were relatively higher in HPV(+) tissues (Figs. 1 and 2), we hypothesized that enhancement of death signaling through TRAILRs could sensitize HPV(+) cells to birinapant’s anti-tumor effect. To establish that a therapeutic agent could enhance the birinapant effects in HPV(+) HNSCC cells, we identified a commercially available anti-TRAILR2/DR5 polyclonal antibody. To test and compare the effects on cell proliferation, we titrated anti-TRAILR2 antibodies to doses of 100, 200, or 400 ng/mL based on published literature and manufacturer’s recommendations, and found that the antibody alone did not produce marked inhibition of cell proliferation compared to control for either UM-SCC-47 (Fig. 5A) or UPCI-SCC-90 (Fig. 5B). However, when combined with birinapant, both cell lines showed a dose-dependent inhibition of cell proliferation as the concentration of anti-TRAILR2 antibody was increased (Fig. 5). Interestingly, increased doses of the antibody did not enhance TNF-α-mediated but increased TRAIL anti-tumor activity, especially in UPCI-SCC-90 cells (Fig. 5). Furthermore, the most robust inhibition of cell proliferation in both cell lines was observed with the triple combination of birinapant, TRAIL, and TRAILR2 antibody (Fig. 5), suggesting a TRAILR2 agonist can augment the effects of TRAIL protein.

### TRAIL and TRAILR2 antibody sensitized UPCI-SCC-90 and UM-SCC-47 cells to birinapant-induced cell cycle alteration and cell death

**Figure 6:**
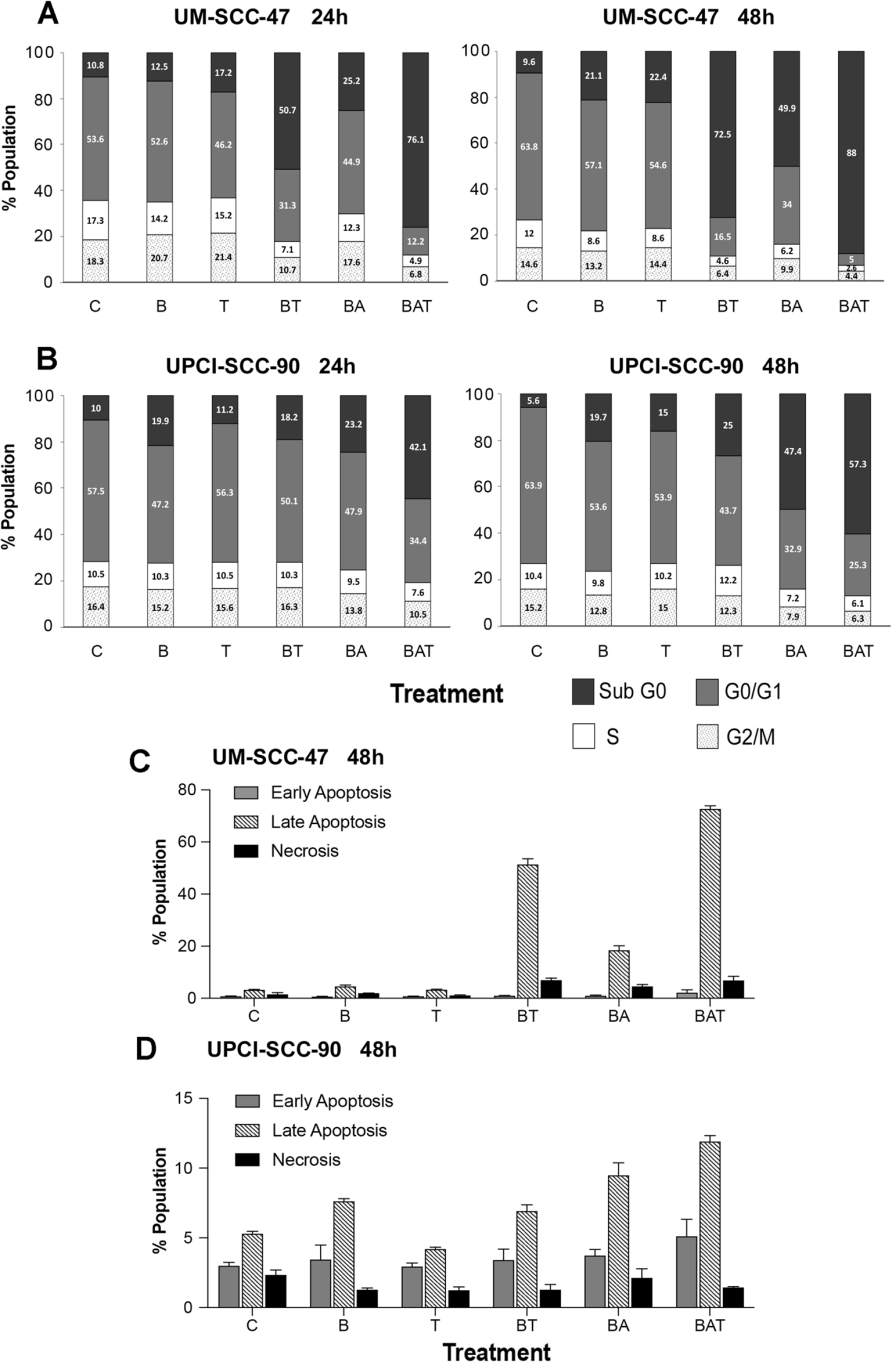
TRAIL and TRAILR2 antibody sensitize UM-SCC-47 and UPCI-SCC-90 cells to birinapant induced cell cycle alteration and cell death. UM-SCC-47 (**A**) and UPCI-SCC-90 cells (**B**) were treated with birinapant (500 nM) or TRAIL (50 ng/mL) alone, combined with TRAILR2 antibody (400 ng/mL), or in triple combination for 24 (left) or 48 h (right). Cells were then stained with propidium iodide (PI) after treatment and analyzed by flow cytometry. The x-axis denotes treatment conditions: *C *control, *B *birinapant, *T *TRAIL, *A *TRAILR2 antibody. BA, BT, and BAT denote combinations of treatments. Percentage of cells in different phases of the cell cycle as presented as a bar graph. UM-SCC-47 (**C**) and UPCI-SCC-90 cells (**D**) were stained with a two-color fluorescent staining followed by flow cytometry to analyze the proportions of early or late apoptotic and necrotic cells. Zombie Violet Fixable Viability Dye was used to identify cells with the damaged plasma membrane in the late apoptosis stage. Anti-phosphatidylserine (PS) antibody was used to identify apoptotic cells exposing PS on their surface at the early apoptosis stage. In all treatment groups, the proportion of apoptosis was greater than that of necrosis. Independent experiments were performed to titrate the drug doses, the combination, and time points previously. Error bars, the standard deviation of 3 replicates from a representative of independent experiments.

In order to evaluate if the observed decrease in cell density was due to cell cycle and/or cytotoxic effects, DNA cytofluorometric analyses of UPCI-SCC-90 and UM-SCC-47 were conducted, after treatments with these drugs alone, or in double or triple combinations (Fig. 6A,B, Supplemental Figures 7, 8). For UM-SCC-47 cells, birinapant or TRAIL alone has minimal effects after 24 h of treatment, and a slightly increased sub-G0 DNA fragmentation was observed at 48 h (Fig. 6A, Supplemental Figure 7A,B). Birinapant plus TRAIL significantly induced sub-G0 DNA fragmentation at 24 h, which was further enhanced by 48 h. While birinapant plus anti-TRAILR2 antibody moderately induced cell death and altered cell cycle, the triple combination treatment induced massive cell death, resulting in 76% and 88% sub-G0 DNA at 24 and 48 h, with severely decreased proportions of both S and G2/M phase DNA (Fig. 6A, Supplemental Figure 7A,B). UPCI-SCC-90 showed a modest increase in sub-G0 DNA when treated with birinapant alone, and weaker effects were observed when treated with TRAIL alone. The proportion of sub-G0 DNA increased when birinapant was combined with TRAIL and increased further when combined with the TRAILR2 antibody in both time points (Fig. 6B, Supplemental Figure 8A,B). A triple combination of birinapant, TRAIL, and TRAILR2 antibody produced the highest sub-G0 DNA accumulation for both cell lines, which is consistent with the findings from the cell density assay. The proportion of cells in the S and G2/M phases did not change significantly except when treated with birinapant plus TRAILR2 antibody or with the triple combination at 48 h for both cell lines (Fig. 6A,B, Supplemental Figures 7B, 8B).

Lastly, Fixed Apoptotic/Necrotic (FAN) flow cytometry analysis was used to discriminate necrotic and apoptotic cells in specific stages. Late stage apoptosis was the primary stage of cell death in both cell lines after 48 h of treatment with single and combination therapies (Fig. 6C,D). UM-SCC-47 cells showed the highest rate of late apoptosis after triple combinatorial treatment, followed by birinapant plus TRAIL combinatorial treatment (Fig. 6C). UPCI-SCC-90 cells also showed highest proportion of late apoptosis in the triple combination group, followed by the birinapant plus TRAIL2-antibody treatment (Fig. 6D).

### Caspase inhibitors reversed apoptosis induced by the combinatorial treatments in UM-SCC-47 and UPCI-SCC-90 cells

**Figure 7:**
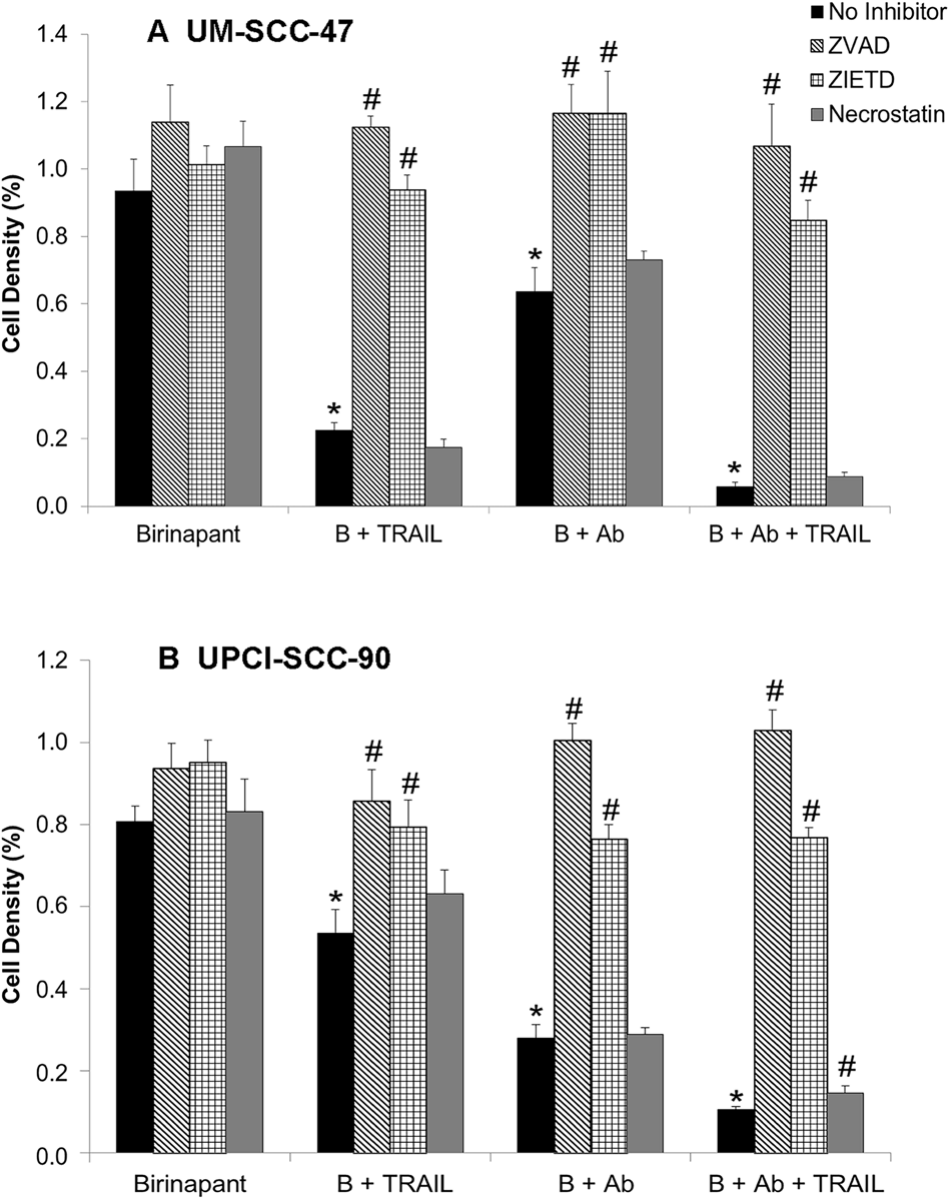
Caspase inhibitors reverse the antiproliferative effect of birinapant and combination treatments. UM-SCC-47 (**A**) and UPCI-SCC-90 cells (**B**) were treated with birinapant alone and combined with TRAIL, TRAILR2 antibody, or a triple combination. Twenty μg/mL of pan-caspase inhibitor ZVAD, caspase-8 inhibitor ZIETD, or RIPK1 inhibitor Necrostatin were added, and cell density was measured by XTT at 72 h for UM-SCC-47 cells and at 120 h for UPCI-SCC-90 cells. Independent experiments were performed to titrate the drug doses, the drug combinations, and time points previously. Error bars, the standard deviation of 6 replicates from a representative of independent experiments. * denotes a significant difference when compared each condition of combinatory treatment with Birinapant alone without caspase or necrosis inhibitors (comparison of other black bars to the first black bar). # denotes the statistical significance showing the caspase and necrosis inhibitors’ effects in each treatment group (other bars compared to the black bars). Student’s *t* test, p < 0.05.

To further analyze the potential mechanism of the combinatorial therapies leading to cell death, we analyzed the effects of pan-caspase (ZVAD), caspase-8 (ZIETD), and RIPK1 (necrostatin) inhibitors on cell density in both UM-SCC-47 and UPCI-SCC-90 cells after treatment by birinapant alone, or in the double or triple combinations, tested at the different time points (Fig. 7 and Supplemental Figure 9). In both cell lines, ZVAD and ZIETD but not Necrostatin reversed the effects of the birinapant plus TRAIL, birinapant plus TRAILR2 antibody, and the triple combinatory treatments. Similar reversal effects were observed in both ZVAD and ZIETD treatment conditions, suggesting the attribution of to the caspase-8**-**dependent apoptosis.

## Discussion

In this study, we investigated the enhanced anti-tumor activity of the death agonist antibody against TRAILR2/DR5 plus TRAIL when combined with the SMAC mimetic IAP inhibitor birinapant in HPV(+) HNSCC cells. Unique genomic and expression signatures in HPV(+) HNSCC tissues were observed, which are distinct from those identified in HPV(−) tumors from TCGA dataset. Most HPV(+) tumors and cell lines rarely exhibited FADD amplification but did have deletions of BIRC2/3 loci on chromosome 11q, while showing TRAIL or TRAILR gene amplification and/or overexpression. These HPV(+) HNSCC lines displayed resistance to birinapant treatment as a single agent in vitro*,* while exhibited sensitivity to the treatment of combined birinapant, TRAIL and TRAILR2 agonist antibody, which mediated death signaling through apoptosis.

We previously showed that overexpression of FADD and BIRC2/IAP1 mRNAs and proteins and their amplifications on chromosome 11q contributed to the cellular sensitivity to birinapant in HPV(−) HNSCC^23^. The genomic amplification of chromosome 11q13 encodes FADD gene, and 11q22 encodes BIRC2/3 genes for IAP1/2 proteins, which are commonly observed in HPV(−), but not in HPV(+) HNSCC (Supplemental Figure 1). Although FADD is not the direct target of birinapant, it plays a critical role in mediating cell apoptosis through the extrinsic death pathway. FADD has been shown to interact with FAS, TNFR, or death receptors (TRAILRs/DRs) by oligomerization and subsequent assembly and activation of caspase-8, which in turn propagates the death signal through pro-apoptotic enzymes including caspases 3/7^27,28^. HNSCC with FADD amplification exhibited worse survival with predominantly conventional surgical treatment, the principle modality from which large specimens were obtained for the cohort of TCGA^23^. In a different cohort, where FADD immunohistochemistry staining was performed in 100 samples of HNSCC tissues, a significant increase in FADD expression in primary tumors was associated with lymph node metastasis and worse disease-free and overall survival^29^. Consistent with the observation in HNSCC, the lung SCC patients with amplification and overexpression of FADD under conventional treatments also had worse disease/progression-free survival from TCGA datasets (Supplemental Figure 3C). This could be explained by the oncogenic function of FADD^30^, or blocking of FADD-induced apoptosis through amplification and overexpression of the anti-apoptosis molecules BIRCs/IAPs, which have been observed in HPV(−) HNSCC and SCCs from other anatomical sites, including the lung, esophagus, cervix and bladder^11^. Overexpression of IAPs in tumor cells inhibit the intrinsic and extrinsic caspases and cell death signaling pathways and induce resistance to death ligands effects mediated through TNF-α and TRAIL receptors^31^. To target IAP proteins, the SMAC mimetics such as birinapant were developed. However, results from early-phase clinical trial testing in human solid tumors showed limited clinical efficacy as a single agent, which indicated that sensitivity to birinapant was not just associated with its target IAP protein expression^12,13^. Rather, we found that the HPV(−) HNSCC cell lines harboring both *FADD* and *BIRC2* amplification and overexpression were more sensitive to birinapant in combination with TNF-α or TRAIL in vitro, as well as in animal models in vivo when combined with radiation, which induces death ligand expression^23^. In contrast to these HPV(−) HNSCC tissues and cell lines, in this study we observed that most HPV(+) HNSCC tissues and cell lines did not exhibit FADD and BIRC2 amplification and mRNA overexpression (Figs. 1, 2, Supplemental Figure 1), although protein expression of cIAP1 and XIAP were abundant (Fig. 3A). These characteristics correlated with their resistance to treatment with birinapant alone and lesser sensitivity with the combination of the death agonists TNF-α when compared with HPV(−) HNSCC cells with FADD amplification (Fig. 4).

In addition, we investigated the genetic alteration rates of *TP53* and *CASP8*, which are known to play important roles in cell apoptosis in the updated HNSCC TCGA cohort^5,24,25^. We observed the differential genetic alterations of *TP53* and *CASP8* between HPV(−) versus HPV(+) HNSCC (Supplemental Figure 2A). In HPV(−) HNSCC, we observed high rates of deleterious mutations of *TP53* and *CASP8,* that are critical in apoptosis and are the pathogenesis and resistance to therapies. The Pickering group showed that *CASP8* is mutated in a subset of HPV(−) HNSCC^24^. *TP53* is also mutated mainly in HPV(−) HNSCC, but with a much lower rate in HPV(+) cancers, where the TP53 protein is inactivated instead by HPV E6 oncoprotein through constant binding and degradation of TP53 protein^32^. In addition, the HPV(+) cell lines used in this study are wildtype *CASP8* and wild type *TP53*^26^, which are consistent with the literature and HNSCC TCGA data. The caspase pathway seems intact in HPV(+) HNSCC cell lines tested in this study, indicating that the drug induced cell death was mainly through the caspase mediated mechanism (Figs. 6, 7), consistent with published literature^5^.

HPV(+) HNSCC tissue and cell lines exhibited additionally altered genomic and expression signatures involved in the death pathway, specifically the amplifications and overexpression of TRAIL and TRAIL receptors. Almost 24% of HNSCC samples exhibited genetic amplifications and overexpression of TRAIL genes on 3q26.31, regardless of HPV status, and TRAIL expression was even higher in HPV(+) tumor tissues and cell lines (Figs. 1, 2). Interestingly, naturally existing TRAIL in the tumor microenvironment of HPV(+) tumors is associated with better prognosis, but fails to prevent tumorigenesis. These tumors lack FADD copy gain and expression, and HPV16 E6 could reduce the protein levels of both FADD and procaspase 8, suppressing the activation of caspases 8, 3, and 2^33^. The low FADD protein expression in most HPV(+) cells supports this hypothesis (Fig. 3A), which could explain why TRAIL or anti-TRAILR2/DR5 alone had minimal inhibitory effects on HPV(+) tumor cells (Figs. 4, 5). In addition, it has been shown that in HPV(+) cervical cancer cell lines, as well as in the keratinocytes transduced with HPV11 or HPV16 E6 and E7 genes, were resistant to treatment with TRAIL and TNFα^34^. Cells expressing HPV oncogenes could perturb TRAIL- and TNFα-mediated death pathways, and instead activate the nuclear factor-κB (NF-κB)-mediated pro-survival pathway^35^. A third mechanism by which HPV(+) tumorigenesis with profound TRAIL expression and intact TRAILR signaling could evade cell death may be explained with the relatively abundant protein expression of IAPs (Fig. 3A), which could block cell death induced through TRAILR signaling. This hypothesis is supported by our observation that IAP1/XIAP inhibition by birinapant sensitized cells to TRAIL plus antibody agonist of TRAILR2/DR5-induced death (Fig. 5).

We observed that there are less samples with genetic deletion and more samples with copy number gain of TRAILRs in HPV(+) HNSCC tissues (Figs. 1, 2), and cell lines expressed a detectable amount of TRAILR2/DR5 surface protein (Fig. 3B,D). The effective anti-tumor activity through birinapant, TRAIL, and TRAILR2/DR5 antibody triple combination observed in this study is consistent with observations from the antibody effect against TRAILR2/DR5 in other cancer types. Abhari et al. studied the cooperative activity of a panel of small-molecule IAP with monoclonal antibodies against TRAIL receptor 1 (Mapatumumab) or TRAIL-R2 (Lexatumumab) to induce apoptosis in neuroblastoma cells. They observed a highly synergistic response between these chemical inhibitors with the antibodies against TRAILRs. They identified receptor-interacting protein 1 (RIP1) as a critical mediator of this synergism through RIP1/FADD/caspase-8 complex-dependent apoptosis^36^. In their study, Necrostatin blocked IAP inhibitor- and TRAIL receptor-triggered apoptosis in neuroblastoma cells, while in our study, ZVAD and ZIETD blocked the triple combination treatment-induced apoptosis, but no blocking effect was observed from Necrostatin (Fig. 7). Allensworth et al. showed that in breast cancer cell lines, SMAC mimetic birinapant induced cell death as a single agent in TRAIL-insensitive SUM190 (ErbB2-overexpressing) cells and significantly increased potency of TRAIL-induced apoptosis in TRAIL-sensitive SUM149 (triple-negative, EGFR-activated) cells^37^. Furthermore, in a study of colorectal HT-29 and pancreatic PANC-1 cells resistant to TRAIL-induced apoptosis, the cells were sensitized to apoptosis by the DR5-specific ligands, which were more effective than DR4-targeted ligands, despite showing a lower cell surface expression of DR5^38^. They speculated that DR5-specific ligands could be superior in amplifying apoptotic signaling, possibly by mediating more effective DR5-triggered downstream apoptotic signaling through rapid kinetics of caspase-8 processing^38^. However, many clinical trials targeting DR4/5 using monoclonal antibodies have not reached the desirable anti-tumor efficacy, which could be due to inhibition of apoptosis at different signaling transduction stages, such as blocking the death-inducing signaling complex (DISC) level by c-FLIP, or more downstream, at the mitochondrial level by overexpression of IAP or Bcl-2 family members, leading to cell death resistance as shown by our and other laboratories^34,39^. To block downstream inhibitory molecules against death signaling, the combined actions of drugs such as birinapant and TRAILR agonists could be a logical step to enhancing the apoptotic signaling mediated by TRAIL and TRAILRs^15^.

In a broader scope that includes other cancer types, a dominant gene amplification pattern of the nine genes surveyed was observed in all five types of SCCs, including those from lung, esophagus, cervical, and bladder, plus ovarian cancers (Supplemental Figure 3A). These findings are consistent with our previous conclusions drawn from the TCGA PanCancer Atlas project, where the strong 11q amplifications were observed in SCCs of head and neck, lung, and esophagus, and overexpression of FADD and BIRC2/3 mRNAs were significantly associated with CNV, especially in HPV(−) SCCs. In addition, *TRAIL* amplification and expression are even higher in cases of lung SCC (46%), as well as 18% of FADD amplification and overexpression. These cancer types along with HNSCC could be suitable for future investigation using combination of birinapant with TRAILR agonist. In our current study, due to the limitation of therapeutic anti-DR5 antibody availability, we were not able to test this combinatorial effect with birinapant in the HPV(+) HNSCC xenograft model in vivo. To test the drug efficacy targeting the death pathways in HNSCC and other SCC tumor models in future studies, and the examination of multiple death molecules indicated in this study using genomic and expression dataset could be informative.

Our genomics and hypothesis driven strategy has been shown to provide predicted targets for combinatorial drug testing for anti-tumor activity in our genetically characterized human HNSCC models that reflect subtypes detected in TCGA. Furthermore, there are multiple therapeutic agents under the development targeting the death receptor pathways, such as multivalent-based antibodies or peptides targeting TRAIL or DR4/5; bi- and tri-specific TRAIL derivatives with single-chain variable fragment (scFv); unconventional and bispecific antibodies targeting DR4/5 and other molecules; chimeric antigen receptors (CARs) targeting TRAILRs; or antibody drug conjugate-like (ADC-like) TRAIL derivatives, to overcome cell resistance to death signaling^6,15^. While eight or more potent and selective monovalent and bivalent SMAC mimetic compounds have been developed to enter the clinical trials for the treatment of cancer, SMAC mimetics compounds exhibited limited clinical activity as single agents^20^. Our study highlights the potential to synergize anti-tumor effects, through the combination of SMAC mimetics, which targets the intrinsic death pathway, with the agents targeting the TRAILRs-mediated extrinsic death pathway.

## Materials and methods

### HPV(+) HNSCC patient samples and bioinformatics analysis of TCGA datasets

TCGA specimens were obtained with informed consent under an IRB approved protocol^11^. Tumor tissue, adjacent normal tissue, and normal whole blood samples were obtained from patients at contributing centers with informed consent according to their local Institutional Review Boards (IRBs, see below). TCGA Project Management has collected necessary human subjects documentation to ensure the project complies with 45-CFR-46 (the “Common Rule”). The program has obtained documentation from every contributing clinical site to verify that IRB approval has been obtained to participate in TCGA. Such documented approval may include one or more of the following: (1) An IRB-approved protocol with Informed Consent specific to TCGA or a substantially similar program. In the latter case, if the protocol was not TCGA-specific, the clinical site PI provided a further finding from the IRB that the already-approved protocol is sufficient to participate in TCGA; (2) A TCGA-specific IRB waiver has been granted; (3) A TCGA-specific letter that the IRB considers one of the exemptions in 45-CFR-46 applicable. The two most common exemptions cited were that the research falls under 46.102(f)(2) or 46.101(b)(4). Both exempt requirements for informed consent, because the received data and material do not contain directly identifiable private information; (4) A TCGA-specific letter that the IRB does not consider the use of these data and materials to be human subjects research. This was most common for collections in which the donors were deceased. The relevant guidelines and regulations were followed during the study.

The complete HNSCC TCGA data were extracted from the latest PanCancer Atlas or Firehose Legacy datasets. Significant focal copy number alterations were extracted from the GISTIC2.0 processing pipeline, and data aggregated on significantly altered lesions are plotted by false discovery rate (FDR) less than 5% and p-values less than 0.05. Differential gene expression analysis of tumor versus normal samples was performed using DeSeq, and all data was log2 transformed (Bioconductor Version 2.12). Genetic altered genes and expression analysis were performed using the online Cancer Genomics cBioportal database (http://www.cbioportal.org/) and presented in Oncoprint, bar graph, and dot plot formats.

### HPV(+) HNSCC cell lines and culture

A panel of eight HPV-positive HNSCC lines, including UM-SCC-47, -104, -105, UD-SCC-2, and 93Vu147T, were provided by Drs. Thomas E Carey, Mark E. Prince, and Carol R Bradford, or described in previous publications^26,40^. UPCI-SCC-90, -152, -154, cells were provided by Drs. Robert Ferris and Susanne Gollin (University of Pittsburgh Medical School)^41^. Authentication of UM-SCC lines was done at the University of Michigan by DNA genotyping of alleles for 9 loci, D3S1358, D5S818, D7S820, D8S1179, D13S317, D18S51, D21S11, FGA, vWA, and the amelogenin locus as described previously^40^. These cell lines were maintained in MEM or DMEM with 10% Fetal Calf Serum (Life Technology, Carlsbad, CA) at 37 °C with 5% CO_2_ for a maximum of 8 weeks. Primary human oral keratinocytes (HOK) were cultured for less than 5 passages, in accordance with the supplier's protocol (Science Cell Research, Carlsbad, CA).

### Drugs, cytokines, and antibody

HPV(+) HNSCC cell lines were titrated with birinapant alone (Tetralogic Pharmaceuticals, Malvern, PA), at varying concentrations, or in combination. The dosing of TNF-α (20 ng/mL), TRAIL (50 ng/mL), and anti-human TRAILR2 polyclonal antibody (400 ng/mL) were used alone and in combination. These reagents were purchased from R&D Systems (Minneapolis, MN).

### Cell proliferation assay

Cells were plated in 96-well plates at predetermined densities for each cell line based on respective doubling times and log-phase growth patterns. Treatment with birinapant 1 µM, TNF-α 20 ng/mL, TRAIL 50 ng/mL, or their combinations occurred 24 h following plating. Cell density was measured using XTT Cell Proliferation Assay Kit from Trevigen (Gaithersburg, MD). Analysis was performed on a microplate reader 72 or 120 h following treatment with drug and/or death agonist. Each cell line and dilution were assayed in six replicates. Half-maximal Inhibitory concentration (IC_50_) was determined after 72 or 120 h using the nonlinear four-parameter regression function in GraphPad Prism (La Jolla, CA). To differentiate between apoptotic and necroptotic cell death, cells were treated with ZVAD, ZIETD, or necrostatin (BD Biosciences, San Jose, CA).

### Western blot analysis

The following primary antibodies were used for Western blotting: polyclonal anti-cIAP-1 (R&D Systems, AF8181), polyclonal anti-cIAP-2 (R&D Systems, AF8171), polyclonal anti-XIAP (R&D Systems, AF8221), and polyclonal anti-FADD (Cell Signaling Technology, 2782). Whole-cell lysates were collected and lysed in NP-40 buffer (Invitrogen, FNN0021) supplemented with 1% Halt protease inhibitor and 1% Halt phosphate inhibitor cocktails from ThermoFisher Scientific (Waltham, MA). Lysates were vortexed and frozen at − 80 °C prior to use. Protein concentrations were determined using the Pierce BCA Protein Assay Kit (ThermoFisher Scientific). Equal amounts of lysates (15 µg) for each cell line were loaded into 4–12% gradient Bis–Tris gels (Invitrogen) and transferred to PVDF membranes for 6 min using the iBlot system (Invitrogen), according to the manufacturer’s standard protocol. Membranes were blocked for one hour in Odyssey blocking buffer (Li-Cor Biosciences, Lincoln, NE) and incubated with the primary antibody overnight at 4 °C. Membranes were washed before incubation with species-appropriate Odyssey secondary antibodies for 1 h at room temperature. Quantification of the protein expression detected by Western blotting was done by protein densitometry normalized to actin with ImageJ (National Institutes of Health, Bethesda, MD).

### Identification of TRAILR2/DR5 protein expression on cell surface

UPCI-SCC-90 and UM-SCC-47 cells were harvested at about 60% confluency using TrypLE Express (Thermo Fisher, 12604013) for 10 min at 37 °C. After collecting and washing, the detached cells were incubated for 30 min at 37 °C to allow for recuperation of surface receptors while minimizing reattachment and cell death. The rested cells were subsequently blocked with Human TruStain FcX (Biolegend, 422301) and stained with PE-conjugated anti-CD262 (TRAILR2/DR5) monoclonal antibody (Thermo Fisher, 12-9908-42). Lastly, the cells were stained with Zombie Violet Fixable Viability Dye (Biolegend, 423113) and were fixed with 2% paraformaldehyde prior to analysis using BD LSRFortessa Cell Analyzer (BD Biosciences). Jurkat cells (ATCC) were stained using the same method as described above and used as a positive control for surface TRAILR2/DR5 expression. The gating used to determine TRAILR2/DR5 surface receptor positivity was based on the population of cells not stained with TRAILR2/DR5 antibody, and the quantification of TRAILR2/DR5 positivity was based only on the live-cell population.

### Cell cycle analysis

UPCI-SCC-90 and UM-SCC-47 cells were plated on 10-cm plates at 1 million cells per plate. Cells were treated 24 h after plating and harvested 48 h after treatment. Cells were counted and stained with propidium iodide (PI) using BD Cycletest Plus DNA Kit prior to analysis on a FACS Canto flow cytometer (BD Biosciences). Data from 10,000 cells per treatment group and time point were analyzed using BD FACSDiva (BD Biosciences) software.

### Apoptosis and necrosis analysis

A two-color fluorescent staining followed by flow cytometry was used to analyze the proportions of early/late apoptotic and necrotic cells. Fixed Apoptotic/Necrotic (FAN) cell test was used with Zombie Violet Fixable Viability Dye (Biolegend, 423113) to identify cells with damaged plasma membrane, and anti-phosphatidylserine (PS) antibody conjugated to Alexa Fluor 488 (Sigma Aldrich, 16-256) to identify apoptotic cells exposing PS on their surface^42^. Cells were fixed with 2% paraformaldehyde prior to analysis using BD LSRFortessa Cell Analyzer (BD Biosciences). Cells treated with 0.1% Triton X prior to staining were used as the positive control for the viability dye as well as the threshold for viability gating.

### Statistical calculations for data analysis

The statistical analysis of % copy number variation (CNV) in HNSCC patients from TCGA datasets was conducted by Fisher exact test. The correlation between CNV and mRNA expression in HNSCC with different HPV status was calculated by Spearman correlation coefficient significance test with p-values presented. The relative gene expression among different normal and tumor groups, DR5 protein expression among the different cell lines detected by the flow cytometry, and data from XTT assays and colorimetric assays were presented as mean ± standard deviation (SD) or standard error (SE), and the statistical significances were calculated by the Student’s *t* test.

### Ethical approval and consent to participate

TCGA specimens were obtained with informed consent under an IRB approved protocol.

### Consent for publication

All authors give consent for the publication of the manuscript in Molecular Cancer.

## Supplementary Information

Supplementary Information.

## Data Availability

The materials and data used and analyzed during the current study are available from the corresponding author on reasonable request.

## Abbreviations

TNF-α: Tumor necrosis factor-α
TRAIL: TNF-related apoptosis-inducing ligand
TNFSF10: Tumor necrosis factor ligand superfamily, member 10
TRAILR2: TNF-related apoptosis-inducing ligand receptor 2
DR5: Death receptor 5
TNFRSF10B: Tumor necrosis factor receptor superfamily, member 10B
HNSCC: Head and neck squamous cell carcinoma
HPV: Human papillomavirus
IAP: Inhibitor of apoptosis proteins
CNV: Copy number variation
TCGA: The Cancer Genome Atlas
ZVAD: Z-VAD-FMK (carbobenzoxy-valyl-alanyl-aspartyl-[*O*-methyl]-fluoromethylketone)
ZIETD: Z-IETD-FMK (Benzyloxycarbonyl-Ile-Glu[OMe]-Thr-Asp[OMe]-fluoromethylketone)
SMAC: Second mitochondria-derived activator of caspases
SCC: Squamous cell carcinomas
FADD: Fas-associated death domain
ADCC: Antibody-dependent cell-mediated cytotoxicity
CDC: Complement-dependent cellular cytotoxicity
FDR: False discovery rate
HOK: Normal human oral keratinocytes
UM-SCC: University of Michigan squamous cell carcinoma
EMEM: Eagle's minimum essential medium
DMEM: Dulbecco’s modified Eagle’s medium
IC_50_: Half-maximal inhibitory concentration
BCA: Bicinchoninic acid
PVDF: Polyvinylidene difluoride
ATCC: American Type Culture Collection
PS: Anti-phosphatidylserine
FAN: Fixed apoptotic/necrotic
PI: Propidium iodide
XTT: 2,3-Bis-(2-methoxy-4-nitro-5-sulfophenyl)-2H-tetrazolium-5-carboxanilide
OD: Optical density
SD: Standard deviation
SE: Standard errors
LR: Larynx
OC: Oral cavity
OP: Oropharynx
RIPK1: Receptor-interacting serine/threonine-protein kinase 1
EGFR: Epidermal growth factor receptor
DISC: Death-inducing signaling complex
scFv: Single-chain variable fragment
CARs: Chimeric antigen receptors
ADC-like: Antibody drug conjugate-like
