# Supplementary material for: Death agonist antibody against TRAILR2/DR5/TNFRSF10B enhances birinapant anti-tumor activity in HPV-positive head and neck squamous cell carcinomas
Source: Sci Rep. 2021 Mar 18;11:6392. doi: 10.1038/s41598-021-85589-5 (PMC7973748; doi:10.1038/s41598-021-85589-5)
Supplement: Supplementary file 1 — Supplementary Information. [file 41598_2021_85589_MOESM1_ESM.pdf]

1/28/2021

Supplemental Information

Death Agonist Antibody against TRAILR2/DR5/TNFRSF10B Enhances Birinapant Anti-Tumor Activity in HPV-Positive Head and Neck Squamous Cell Carcinomas

Yi An[1], Jun Jeon[1,2], Lillian Sun[1], Adeeb Derakhshan[1], Jianhong Chen[1], Sophie Carlson[1], Hui Cheng[1], Christopher Silvin[1], Xinping Yang[1], Carter Van Waes[1,*], Zhong Chen[1,*]

1. Tumor Biology Section, Head and Neck Surgery Branch, National Institute on Deafness and Other Communication Disorders, National Institutes of Health, Bethesda, MD 201892

2. NIH Medical Research Scholars Program, Bethesda, Maryland.

* Contributed equally as senior authors.

Correspondence: Zhong Chen, MD, PhD; Phone: 301-435-2073; Fax: 301-402-4216; E-mail: chenz@nidcd.nih.gov. Carter Van Waes, MD, PhD; Phone: 301-402-4216; Fax: 301-402-1140; E-mail: vanwaesc@nidcd.nih.gov. Head and Neck Surgery Branch, NIDCD/NIH, Building 10, 7N240, Bethesda, MD 20892.

Supplemental Figure Legends

Supplemental Figure 1. Chromosome view of death genes in HPV(+) HNSCC from TCGA datasets and HPV(+) HNSCC cell lines

Copy number variation (CNV) of FADD, BIRC2/3, TNFRSF10A/B/C/D in 80 HPV(+) HNSCC tissues from TCGA Pan Cancer Atlas dataset (left panels) and 11 HPV(+) HNSCC cell lines (right panels) are shown by chromosome view. (A) chromosome 11q13/11q22 for FADD and BIRC2/3 loci, indicated by red bars; (B) chromosome X for XIAP loci; (C) 3q26.31 for TRAIL/TNFSF10 loci; (D) chromosome 8p21.3 for TNFRSF10A/B/C/D loci. Heatmaps show the quantification of segmented CNV; red, gain; blue, loss; and white, no change.

Supplemental Figure 2. Genetic alterations of TP53 and CASP8 in HNSCC TCGA cohort

(A) Oncoprint presents genetic alteration of *TP53* and *CASP8* in HNSCC from TCGA Pan Cancer Atlas dataset. (B) The mutual exclusivity of *TP53* with *CASP8* genetic alterations is observed with statistical significance.

Supplemental Figure 3. Significant genetic and expression alterations of molecules involved in death pathways in other cancer types from TCGA datasets

(A) The copy number variation and mutations of the genes involved in the death pathways were ranked across all cancer types from TCGA Pan Cancer Atlas datasets, and the top cancers with the highest frequency of genetic alterations were presented in x axis. Percentages of the genetic alterations are presented in y axis. Red: gene amplification, blue: gene deletion, green: mutation, gray: multiple genetic variations. (B) Genetic and expression alterations of genes involved in death pathways were identified in the TCGA lung SCC database from the c-Bioportal website and presented in Oncoprint. Percentage of each gene's alteration in total patient samples was represented and each bar represented as individual patient sample. Lung SCC patients with FADD

amplification exhibited worse disease/progression free survival (C), while patients with amplification or overexpression of TNFSF10/TRAIL had a better survival (D).

Supplemental Figure 4. Full images of the Western blots for FADD, IAPs and TRAILR2/DR5 protein expression in HPV(+) HNSCC cell lines

The original Western blot images of protein expression from whole cell lysates of HOK and a panel of HPV(+) HNSCC cell lines are presented. The arrows indicate the protein bands detected by Western blots used in the Figure 3A.

Supplemental Figure 5. Protein expression of TRAILR2/DR5 in HPV(+) HNSCC cell lines

UM-SCC47 (top row) and UPCI-SCC-90 cells (second row) were stained with fluorescent conjugated anti-TRAILR2/DR5 antibody (X-axis) and Zombie-violet viability dye (Y-axis), and the staining intensity is presented by dot plots. The left column shows staining with viability dye only, and two gates each delineate Zombie-violet viability dye and TRAILR2/DR5 positivity. The same gating was applied for analyzing TRAILR2/DR5 stained samples in the second column. TRAILR2/DR5 staining of Jurkat cells (bottom row) was served as a positive.

Supplemental Figure 6. The anti-proliferative effects of birinapant, TNF, and TRAIL alone and in combination in HPV(+) cell lines at day 5

Effect of birinapant with and without death agonists TNFα or TRAIL on 8 HPV(+) cell lines. Percent growth inhibition as assessed by XTT proliferation assay on day 5 post treatment with birinapant 1 μM, TNFα 20 ng/mL (top), TRAIL 50 ng/mL (bottom), or the combinations. Independent experiments performed to obtain non-treated and birinapant controls for comparison with TNFα, TRAIL, or their combination with birinapant. Values normalized to non-treated cells for the same experiment. Non-treated and birinapant controls for comparison with TNFα or TRAIL or their combinations. Error bars, standard deviation of 6 replicates. Student's T-Test: *

$p<0.05$. Columns marked with * were statistically significantly different than the untreated control. Separately, a bar overlying the * indicates a statistically significant difference between combination treatment and the individual treatment(s) that the bar extends to.

Supplemental Figure 7. TRAIL and TRAILR2 antibody sensitize UM-SCC-47 cells to birinapant induced cell cycle alteration and cell death

UM-SCC-47 were treated with birinapant (500 nM) or TRAIL (50 ng/mL) alone, combined with TRAILR2 antibody (400ng/mL), or in triple combination. Cells were then stained with propidium iodide (PI) 24 hours (A) or 48 hours (B) after treatment, and analyzed by flow cytometry. Histogram presents the percentage of live cells in different phases of cell cycle (G1, S, G2/M) and dead cells (SubG0).

Supplemental Figure 8. TRAIL and TRAILR2 antibody sensitize UPCI-SCC-90 cells to birinapant induced cell cycle alteration and cell death

UPCI-SCC-90 cells were treated with birinapant (500 nM) or TRAIL (50 ng/mL) alone, combined with TRAILR2 antibody (400ng/mL), or in triple combination. Cells were then stained with propidium iodide (PI) 24 hours (A) or 48 hours (B) after treatment and analyzed by flow cytometry. Histogram presents the percentage of live cells in different phases of cell cycle (G1, S, G2/M) and dead cells (SubG0).

Supplemental Figure 9. Antiproliferative effect of birinapant and combination treatments is reversed by caspase inhibitors

UM-SCC-47 (A) and UPCI-SCC-90 cells (B) were treated with birinapant alone and in combination with TRAIL, TRAILR2 antibody, or in triple combination. Twenty mg/mL of pan-caspase inhibitor ZVAD, caspase-8 inhibitor ZIETD, or RIPK1 inhibitor Necrostatin were added, and cell density was measured by XTT at 120 hours for UM-SCC 47 cells, and at 72 hours for

UPCI-SCC 90 cells. * and # denotes a significant p-vale of < 0.05 by two tailed student's t-test when compared to no treatment controls and no inhibitor controls in 6 replicates respectively.

Supplemental Table 1. Titrate birinapant IC50 alone or combination effects in HPV(+) HNSCC cell lines

XTT Cell Proliferation Kits were used to determine birinapant IC50 alone or combination effects in a panel of HPV (+) HNSCC lines. Cells were plated in 96-well plates and treated with a serial dilution of birinapant alone (1nM-5000nM), or in combination with 20 ng/mL TNFα or 50ng/ml TRAIL. Inhibitory concentration 50% (IC50) was determined on day 3 or day 5 using the nonlinear four-parameter regression function in GraphPad Prism (La Jolla, CA).

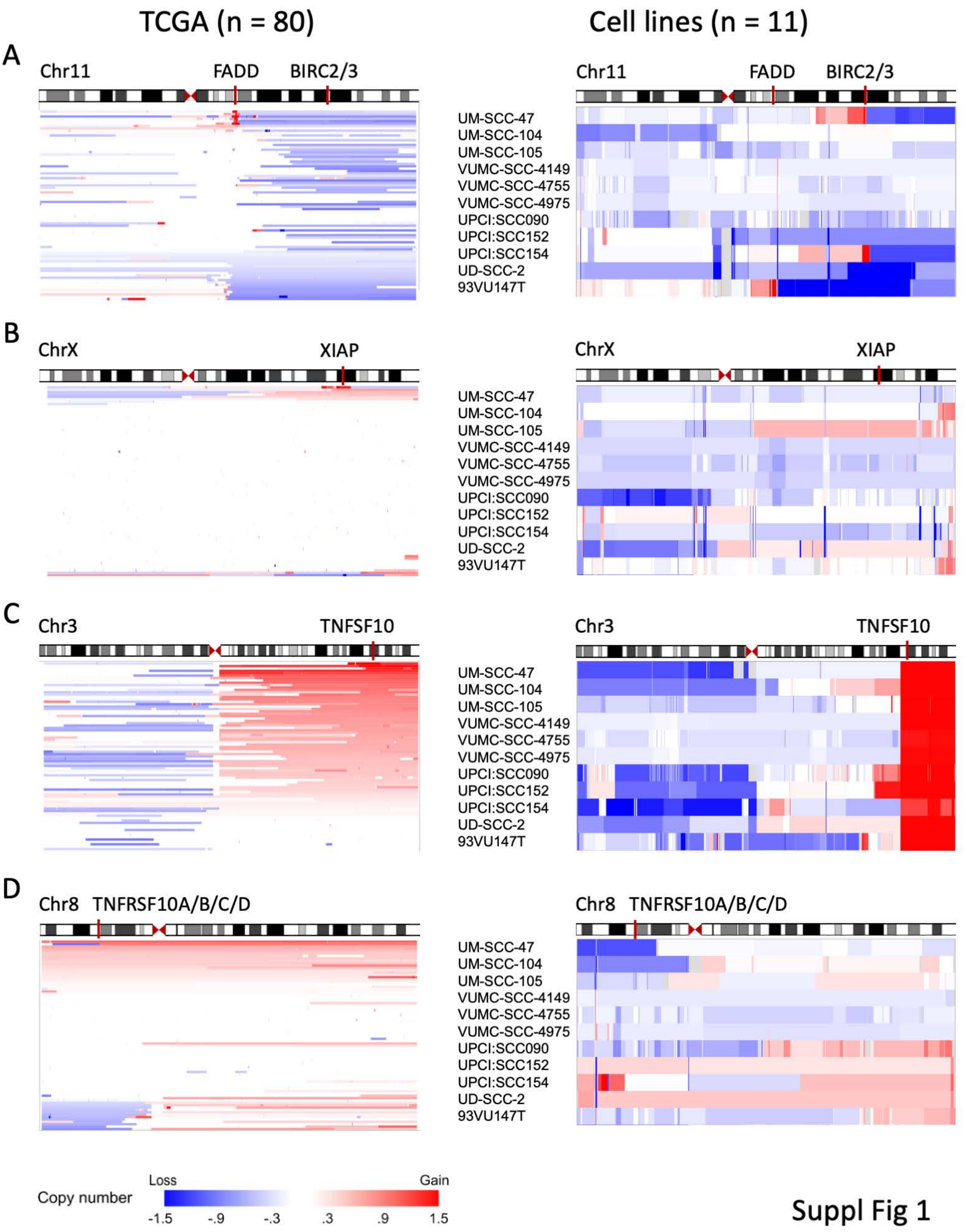
TCGA (n = 80)
Cell lines (n = 11)
A
Chr11
FADD
BIRC2/3
UM-SCC-47
UM-SCC-104
UM-SCC-105
VUMC-SCC-4149
VUMC-SCC-4755
VUMC-SCC-4975
UPCI:SCC090
UPCI:SCC152
UPCI:SCC154
UD-SCC-2
93VU147T
B
ChrX
XIAP
C
Chr3
TNFSF10
D
Chr8
TNFRSF10A/B/C/D
Copy number
Loss
Gain
-1.5
-.9
-.3
.3
.9
1.5

Suppl Fig 1

A

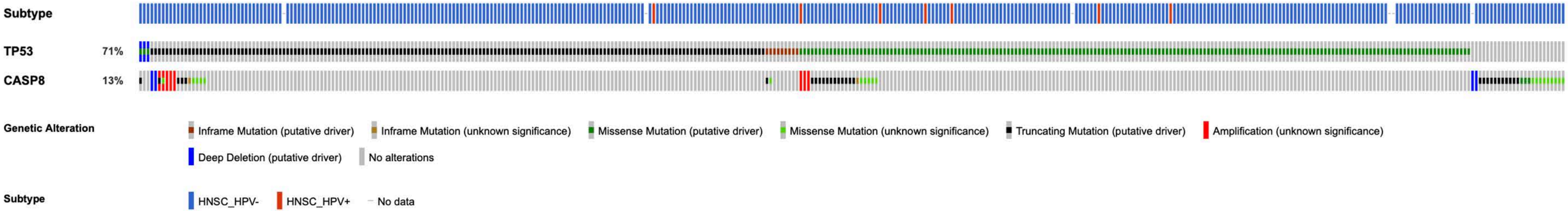

B

A	B	Neither	A Not B	B Not A	Both	Log2 Odds Ratio	p-Value	q-Value ▲	Tendency
**TP53**	**CASP8**	118	314	25	39	-0.770	0.039	**0.039**	**Mutual exclusivity**

Suppl Fig 2

A

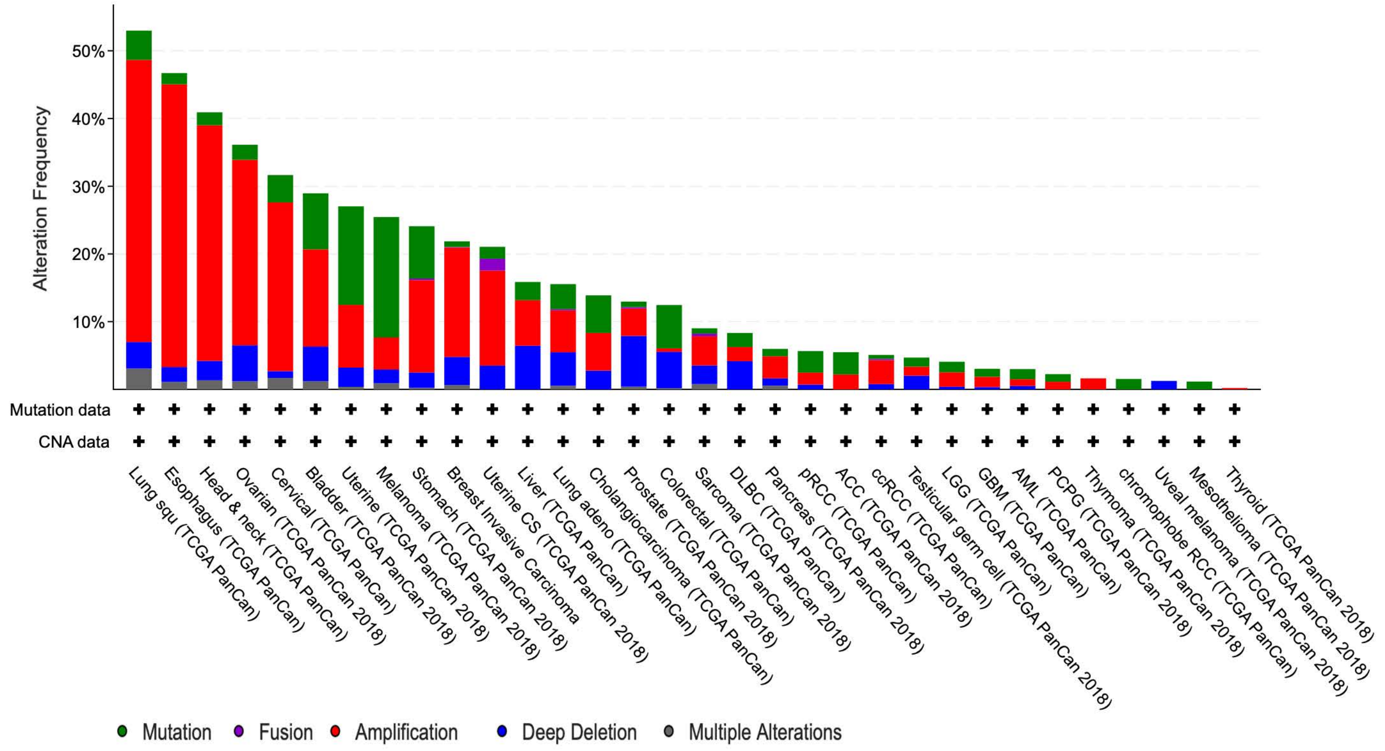

B

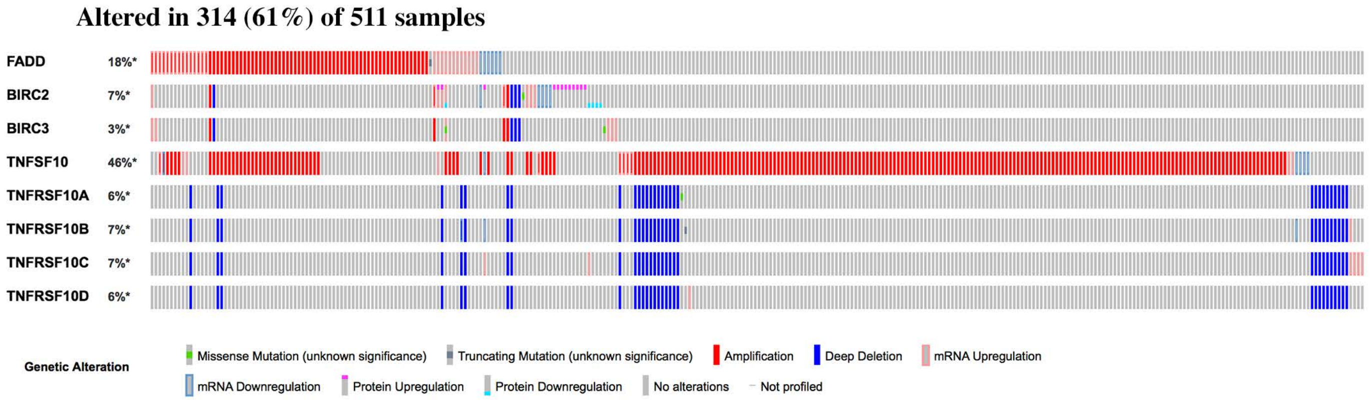

C

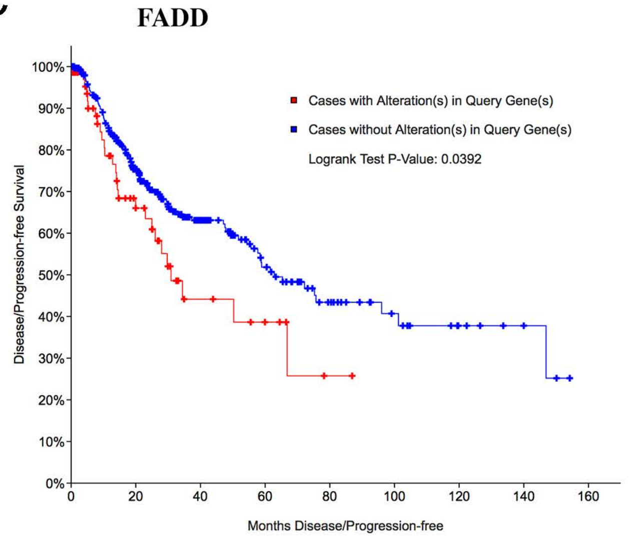

	Number of Cases, Total	Number of Cases, Relapsed/Progressed	Median Months Disease-free
Cases with Alteration(s) in Query Gene(s)	**71**	**27**	**30.88**
Cases without Alteration(s) in Query Gene(s)	**306**	**106**	**62.81**

D

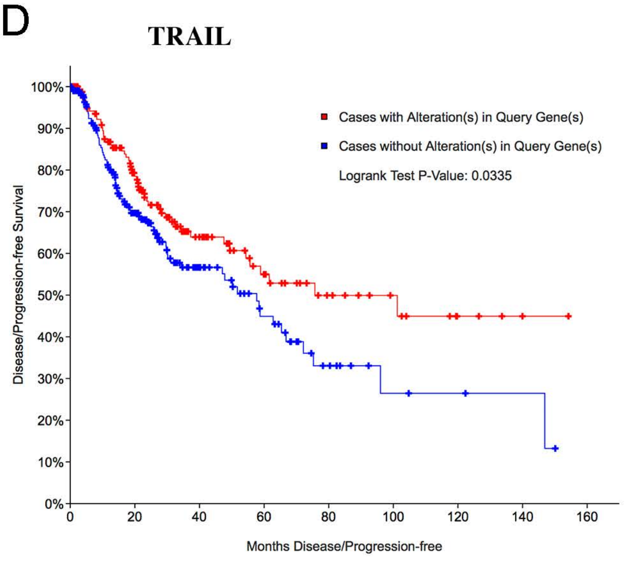

Number of Cases, Total	Number of Cases, Relapsed/Progressed	Median Months Disease-free
**172**	**54**	**75.72**
**205**	**79**	**57.72**

Suppl Fig 3

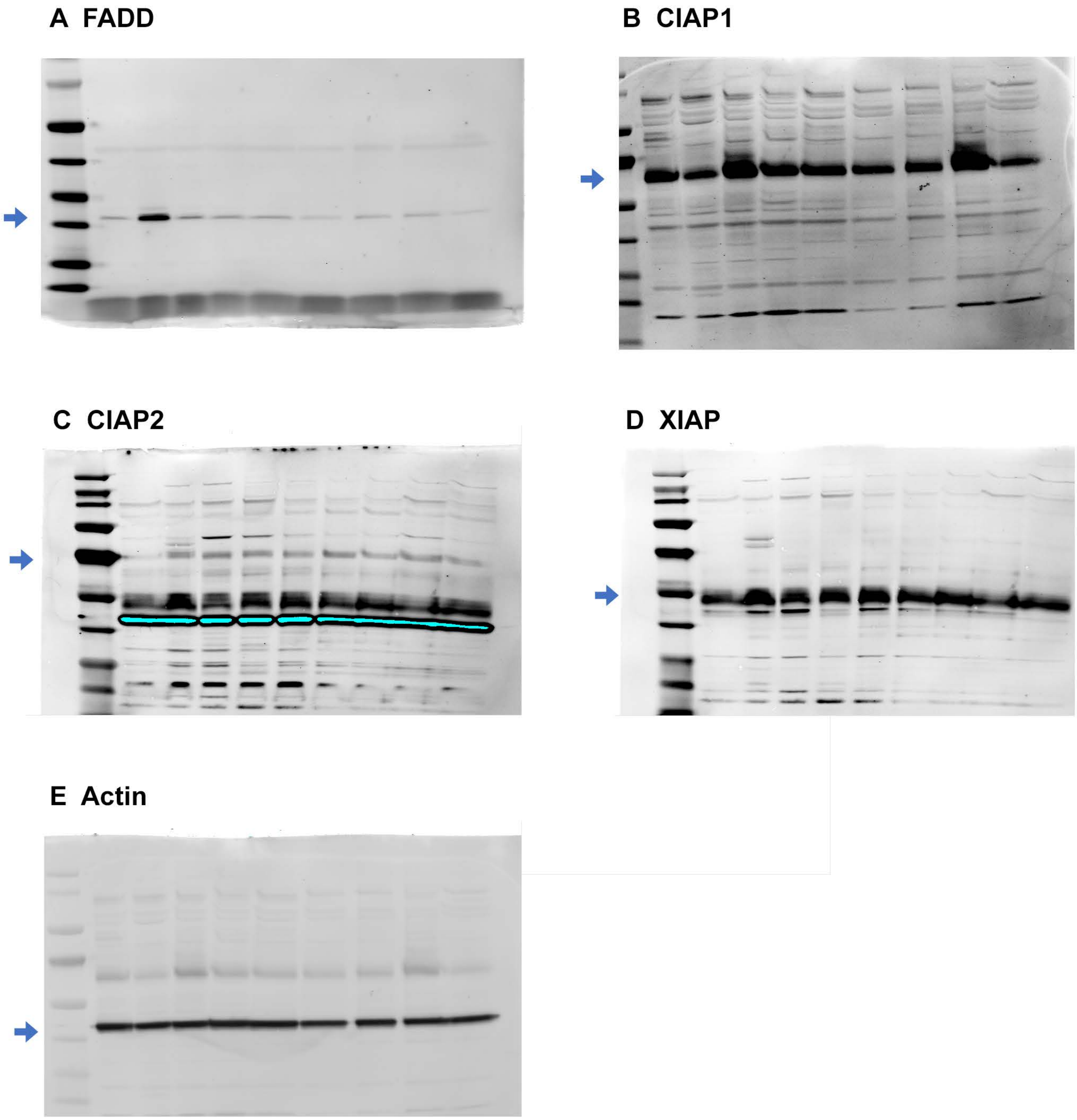

Suppl Fig 4

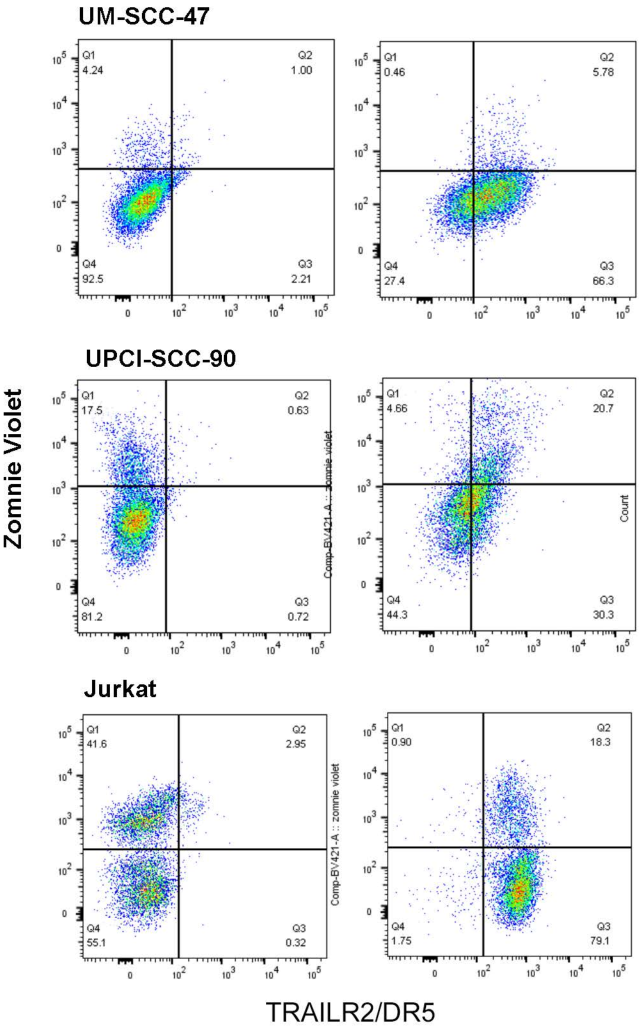
UM-SCC-47
Q1 4.24
Q2 1.00
Q4 92.5
Q3 2.21
Q1 0.46
Q2 5.78
Q4 27.4
Q3 66.3
UPCI-SCC-90
Q1 17.5
Q2 0.63
Q4 81.2
Q3 0.72
Q1 4.66
Q2 20.7
Q4 44.3
Q3 30.3
Comp-BV421-A :: zomnie violet
Count
Jurkat
Q1 41.6
Q2 2.95
Q4 55.1
Q3 0.32
Q1 0.90
Q2 18.3
Q4 1.75
Q3 79.1
Comp-BV421-A :: zomnie violet
Zomnie Violet
TRAILR2/DR5

Suppl Fig 5

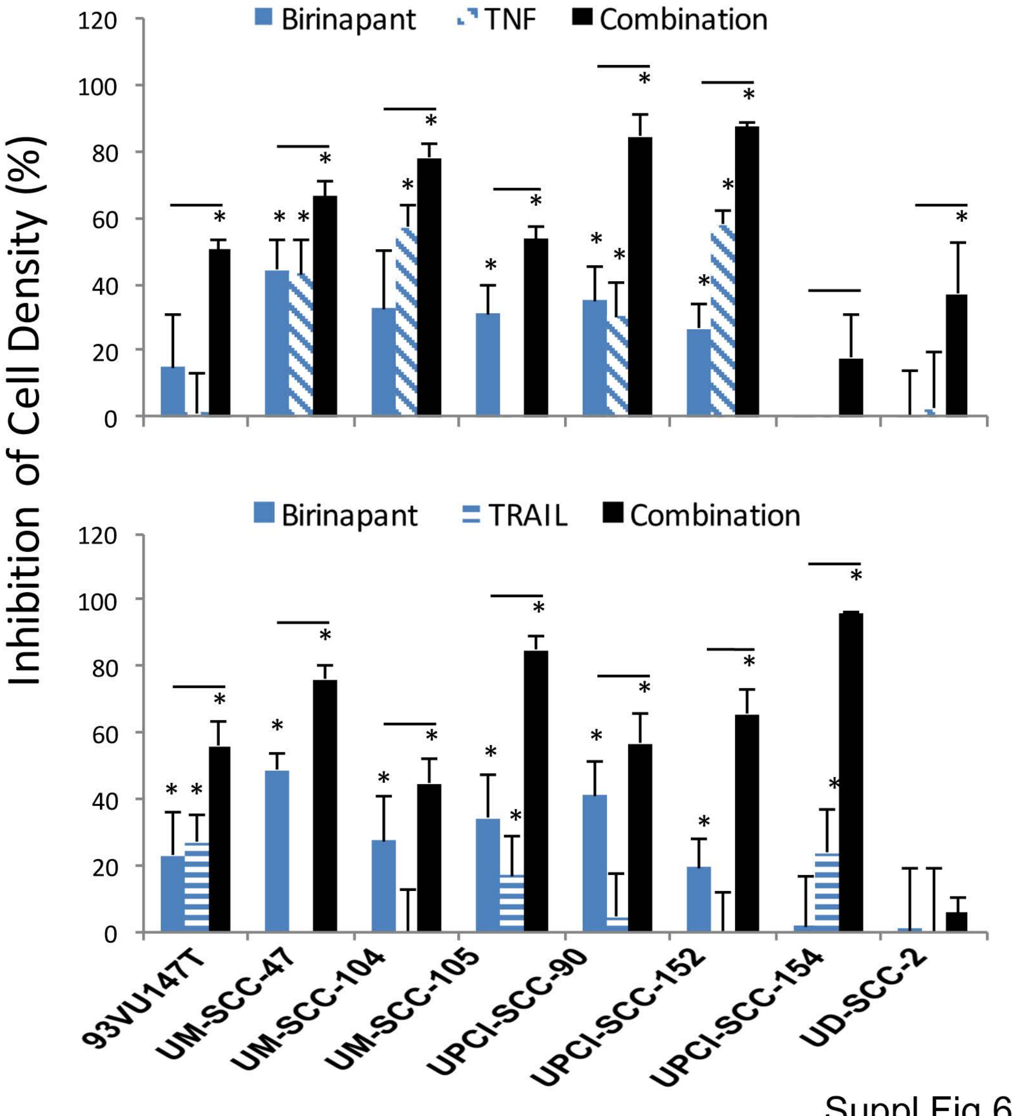
Birinapant
TNF
Combination
Birinapant
TRAIL
Combination
Inhibition of Cell Density (%)
0
20
40
60
80
100
120
93VU147T
UM-SCC-47
UM-SCC-104
UM-SCC-105
UPCI-SCC-90
UPCI-SCC-152
UPCI-SCC-154
UD-SCC-2

Suppl Fig 6

A UM-SCC-47 24 hours

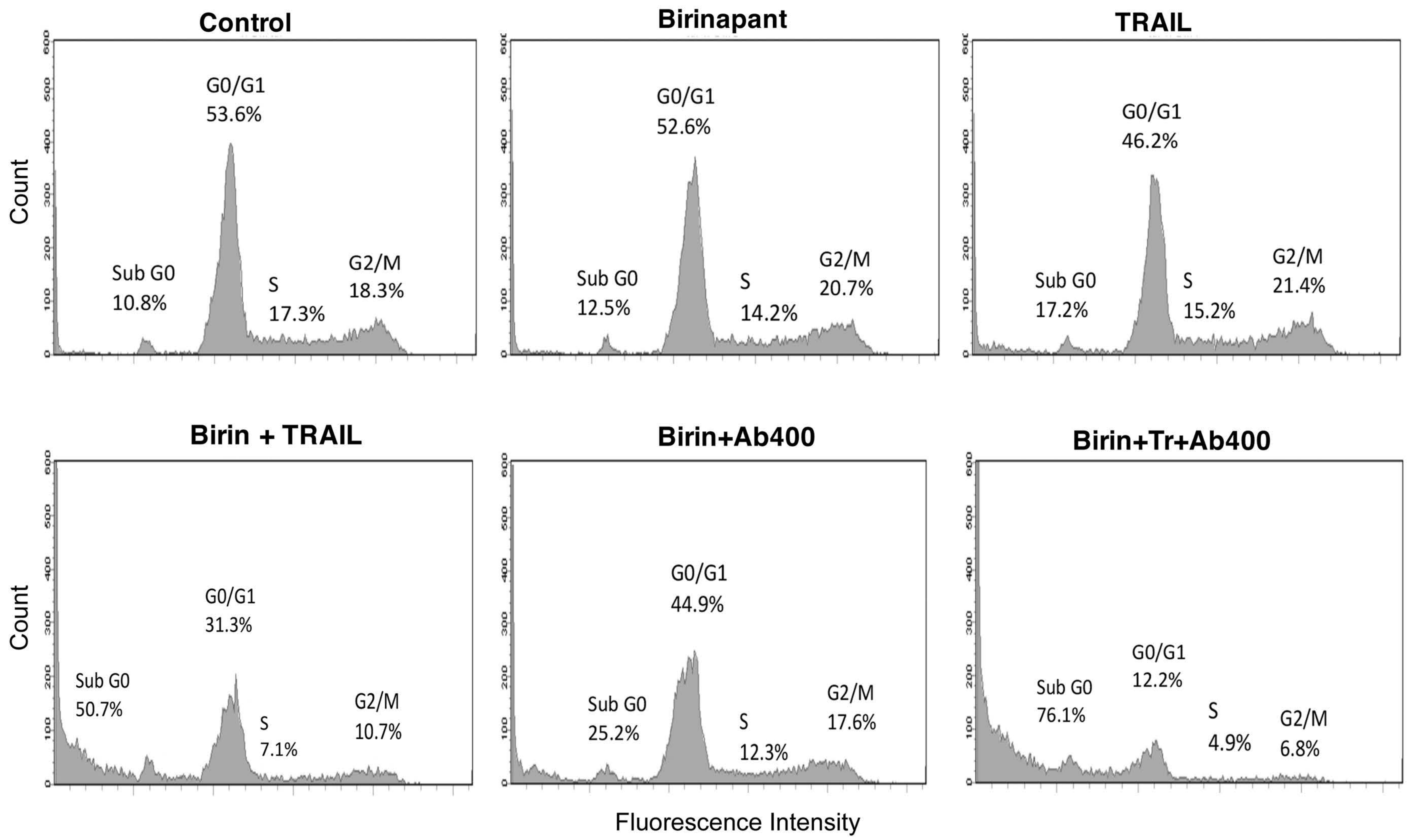

B UM-SCC-47 48 hours

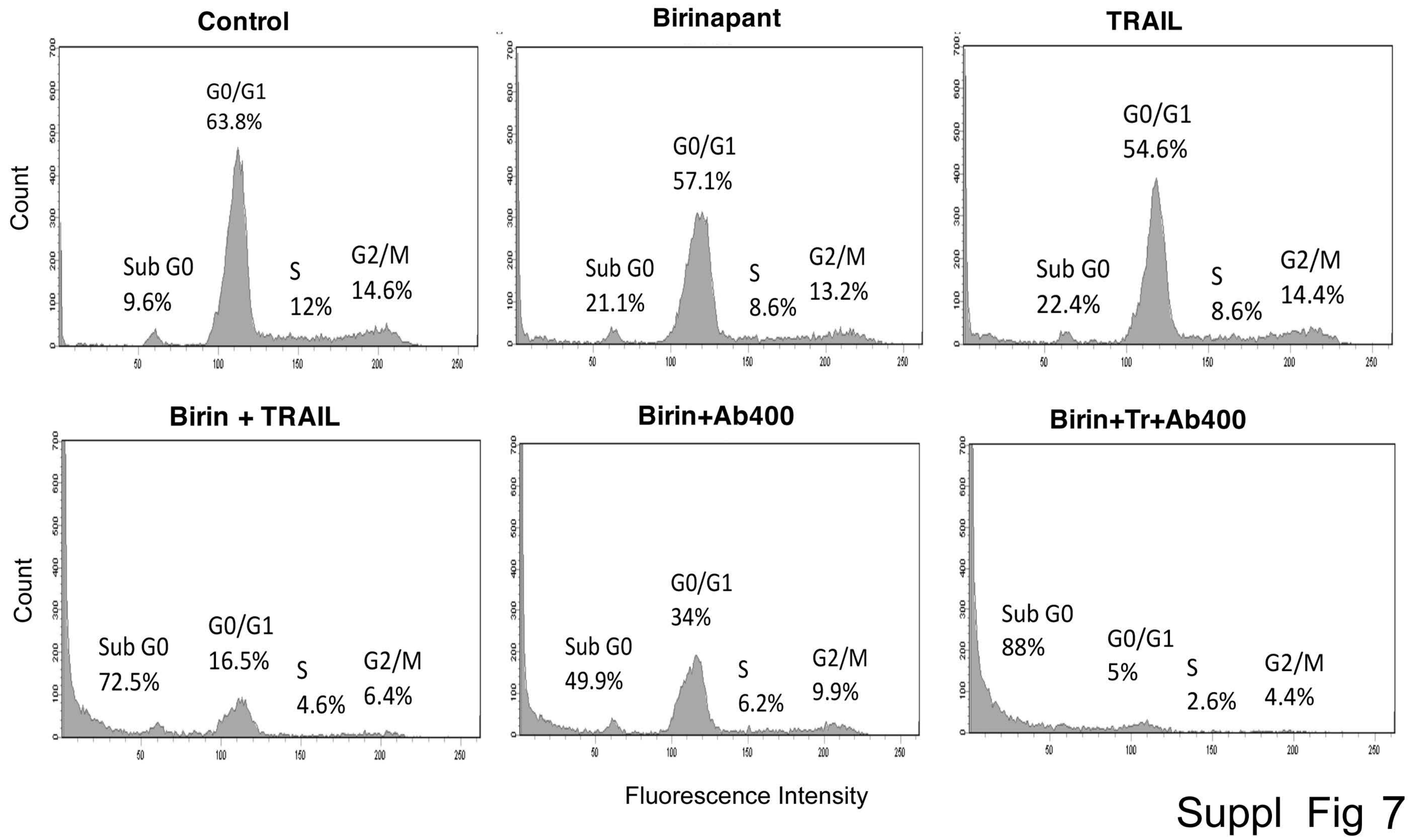

Suppl Fig 7

A UPCI-SCC-90 24 hours

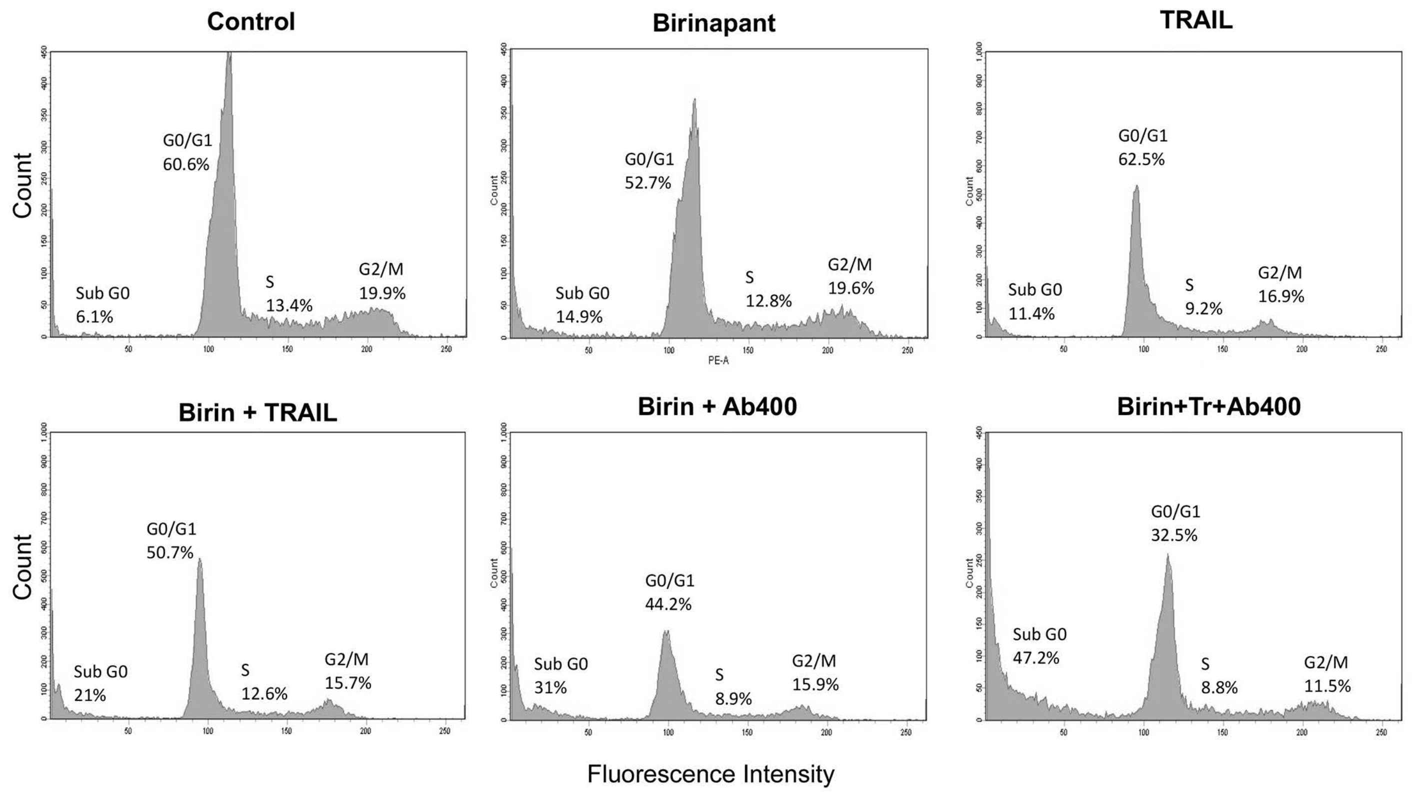

B UPCI-SCC-90 48 hours

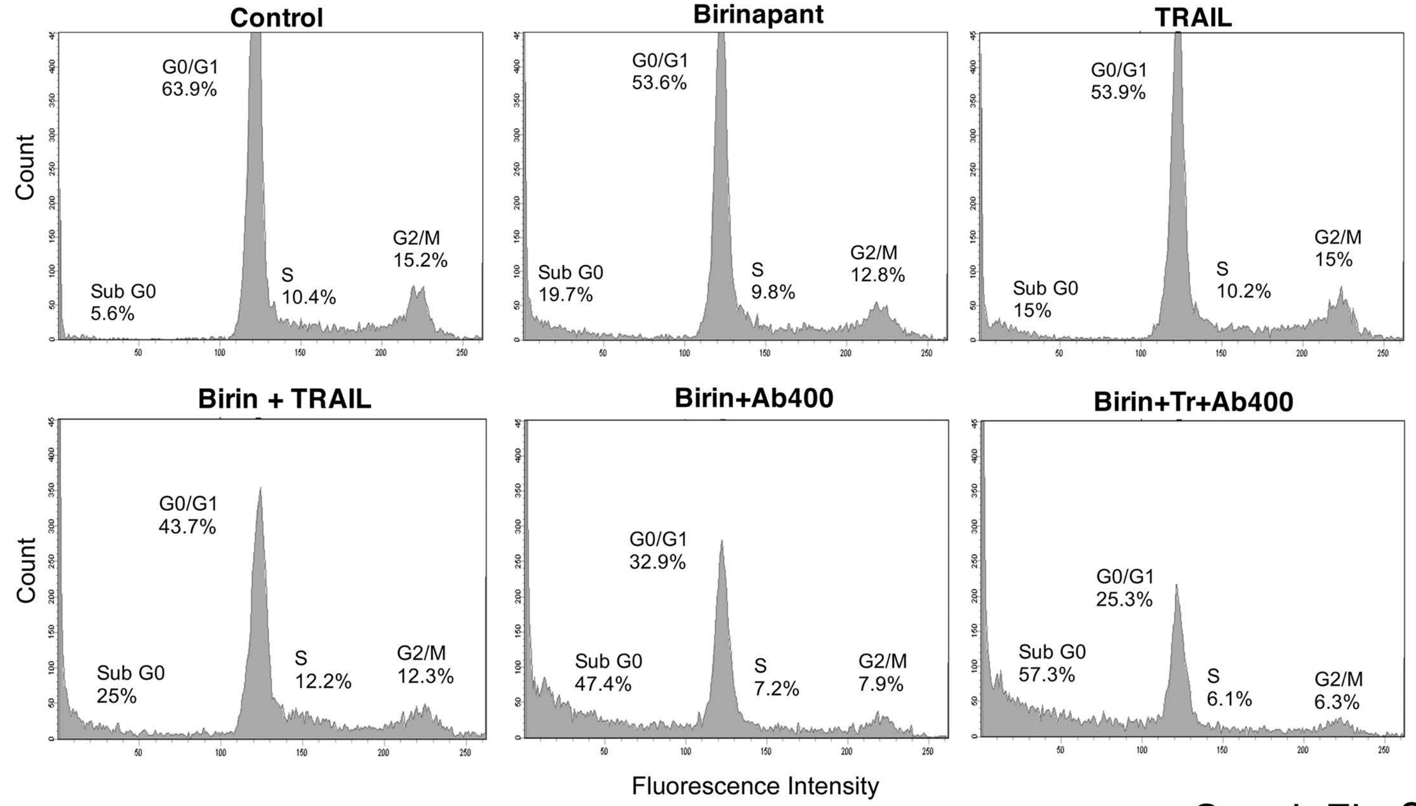

Suppl Fig 8

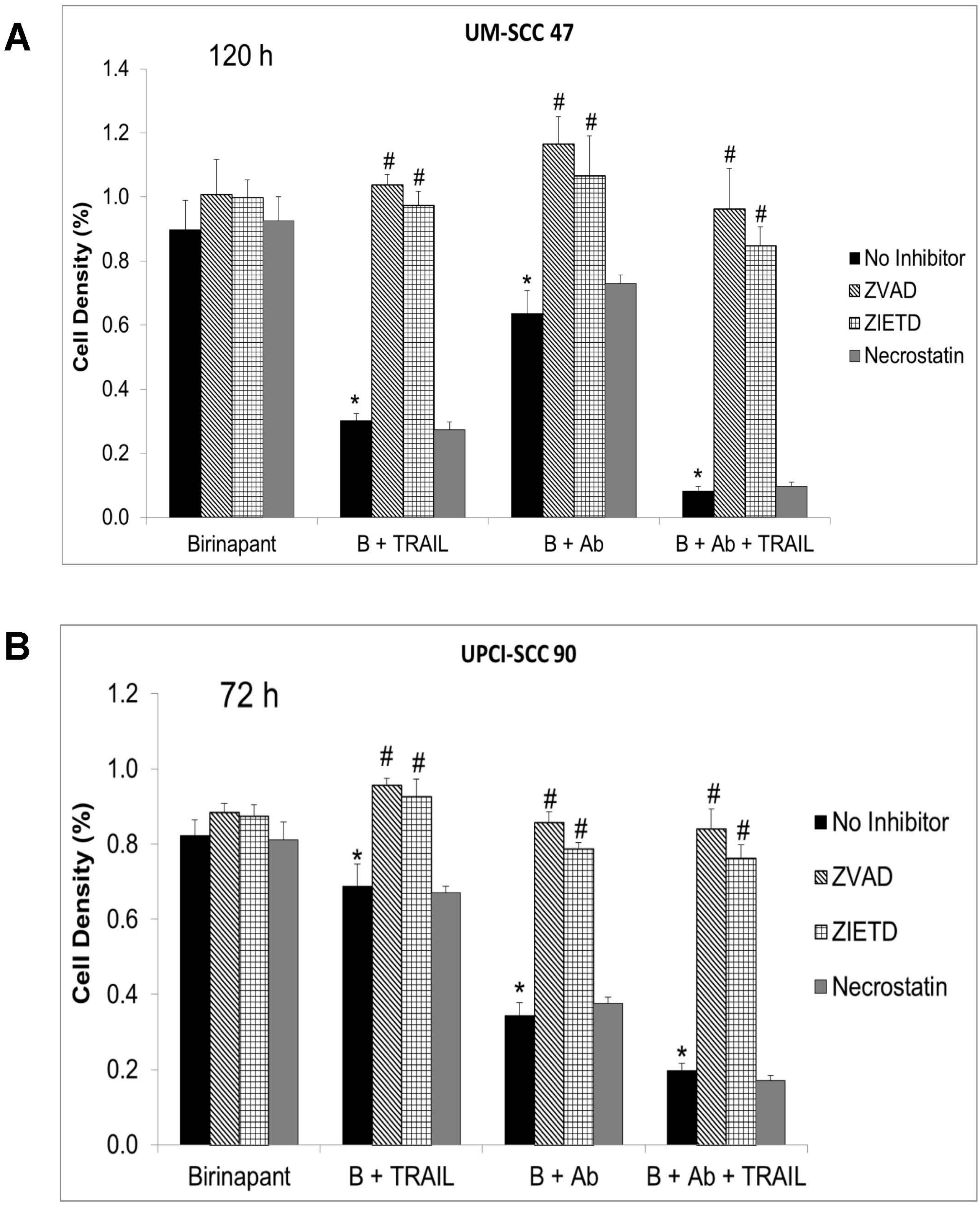
A
UM-SCC 47
120 h
Cell Density (%)
1.4
1.2
1.0
0.8
0.6
0.4
0.2
0.0
Birinapant
B + TRAIL
B + Ab
B + Ab + TRAIL
No Inhibitor
ZVAD
ZIETD
Necrostatin
B
UPCI-SCC 90
72 h
Cell Density (%)
1.2
1.0
0.8
0.6
0.4
0.2
0.0
Birinapant
B + TRAIL
B + Ab
B + Ab + TRAIL
No Inhibitor
ZVAD
ZIETD
Necrostatin

Suppl Fig 9

Supplemental Table 1. IC50 of Birirnapant in HPV(+) HNSCC cell lines

IC_{50} (nM) – 3 Days Post Treatment

Cell Line	Birinapant	Birinapant + TNF	Birinapant + TRAIL
93VU147T	>5000	200	100
UM-SCC-47	>5000	20	32
UM-SCC-104	>5000	2.5	>5000
UM-SCC-105	>5000	>5000	2
UPCI-SCC-90	>5000	5.6	>5000
UPCI-SCC-152	>5000	1.1	8.9
UPCI-SCC-154	>5000	>5000	0.4
UD-SCC-2	>5000	794	>5000

IC_{50} (nM) – 5 Days Post Treatment

Cell Line	Birinapant	Birinapant + TNF	Birinapant + TRAIL
93VU147T	>5000	177	126
UM-SCC-47	500	1.6	28
UM-SCC-104	>5000	0.3	>5000
UM-SCC-105	631	661	3.2
UPCI-SCC-90	>5000	1	25
UPCI-SCC-152	>5000	0.4	10
UPCI-SCC-154	>5000	>5000	0.6
UD-SCC-2	>5000	1995	>5000

IC50 values (nM) for UM-SCC cell lines treated with birinapant (1 nM– 5000 nM) -/+ TNF-α or TRAIL. Cell lines that did not demonstrate at least 50% growth inhibition for a given condition are indicated with IC50 > 5000 nM.
